# Supplementary material for: Structural Basis of Vesicle Formation at the Inner Nuclear Membrane
Source: Cell. 2015 Dec 17;163(7):1692–701. doi: 10.1016/j.cell.2015.11.029 (PMC4701712; doi:10.1016/j.cell.2015.11.029)
Supplement: Document S1. Supplemental Experimental Procedures [file mmc1.pdf]

Cell

Supplemental Information

Structural Basis of Vesicle Formation at the Inner Nuclear Membrane

Christoph Hagen, Kyle C. Dent, Tzviya Zeev-Ben-Mordehai, Michael Grange, Jens B. Bosse, Cathy Whittle, Barbara G. Klupp, C. Alistair Siebert, Daven Vasishtan, Felix J. B. Bäuerlein, Juliana Cheleski, Stephan Werner, Peter Guttmann, Stefan Rehbein, Katja Henzler, Justin Demmerle, Barbara Adler, Ulrich Koszinowski, Lothar Schermelleh, Gerd Schneider, Lynn W. Enquist, Jürgen M. Plitzko, Thomas C. Mettenleiter, and Kay Grünewald

SUPPLEMENTAL EXPERIMENTAL PROCEDURES

Electron Cryo-Microscopy of Vitreous Sections - CEMOVIS

CEMOVIS is the 'gold standard' among sectioning methods for electron microscopy (Bleck et al., 2010; Gunkel et al., 2015; Mielanczyk et al., 2014). However, structural artefacts such as inhomogeneous compression and membrane crevasses (Figure 2B, asterisks) (Al-Amoudi et al., 2005; Bouchet-Marquis and Hoenger, 2011; Pierson et al., 2011), impaired higher-resolution analysis.

Compression due to the sectioning process was estimated based on the change in aspect ratio of HSV-1 capsids. A non-linear compression dependency on the nominal section feed similar to that described in (Han et al., 2008) was observed. Projection images from vitreous sections were corrected for this thickness-dependent compression along the cutting direction (knife marks) by rescaling and interpolation, and were low-pass filtered for an improved signal to noise ratio. The projection images of tomographic tilt series were aligned using a MATLAB-implementation (provided by Matthias Eibauer, University of Zurich, Department of Biochemistry, Switzerland) of marker-free image registration algorithms described in (Sorzano et al., 2009), in combination with the global alignment correction capabilities of IMOD (Kremer et al., 1996; Mastronarde, 2008) to account for electron beam-induced movements of the vitreous section (Hsieh et al., 2006). Tomographic reconstructions were corrected for compression according to the model given in (Al-Amoudi et al., 2005), including lateral re-positioning of all X/Y-slices in the Z-direction, using MATLAB scripts provided by Lars-Anders Carlson (University of California, Department of Molecular & Cell Biology, Berkeley, CA). If not stated otherwise, CEMOVIS tomograms presented here are binned with a kernel of 2x2x2 and were, additionally, 3D-Gaussian-filtered (Amira 5.2; FEI, Eindhoven, The Netherlands). Slices are shown in the corresponding voxel size thickness.

Live Cell Three-Dimensional Structured Illumination Microscopy - 3D-SIM

Our setup was equipped with a manufacturer-supplied apparatus and objective heater to allow medium-term imaging under standard live cell conditions (37 °C and 5 % CO_2). Previous live-samples assessed by 3D-SIM have concentrated on bacterial studies at 30 ºC (Strauss et al., 2012; Turnbull et al., 2014) or for HeLa cells at 24 ºC (Shao et al., 2011). The OMX V3 Blaze system parameters were adjusted for temperature-dependent changes in immersion oil refractive index with single layers of TetraSpeck fluorescent microspheres (Life Technologies), and optical transfer functions (OTFs) were generated from individual 110 nm diameter 505/515 fluorescent microspheres (Life Technologies). Phase parameters were adjusted for optimal imaging at 488 nm, and data was acquired using an appropriately matched immersion oil. 3D-SIM data were acquired with a Z-distance of 125 nm and with 15 images per plane (five phases, three angles). The raw data was computationally reconstructed using a Wiener (high-frequency) filter setting of 0.002 and channel- and temperature-specific OTFs employing the softWoRx 6.0 software package (GE Healthcare) to obtain a super-resolution 3D image stack of 32-bit depth, with a pixel size of 40x40 nm. Typically nuclei imaged were in between 6 µm and 12 µm in height. The resolution achieved was ~120 nm in the lateral direction and ~300 nm in the axial direction. The reconstructed data was thresholded for each channel to the stack modal grey value (representing the centre of the background intensity level) and converted to 16-bit tiff-stacks.

Correlative Fluorescence and Soft X-Ray Cryo-Microscopy/Tomography - CryoXM/T - at the HZB TXM at Beamline U41-FSGM of the BESSYII Electron Storage Ring in Berlin/Germany

Incubation of the BK cells on special grids for the Helmholtz-Zentrum Berlin full-field transmission soft X-ray microscope (HZB TXM), live-cell light microscopy, cryo-immobilization by plunge-freezing and correlated fluorescence/cryoXM/T are detailed in (Hagen et al., 2012) and (Hagen et al., 2014). For methodological reviews, see (Dent et al., 2014; Schneider et al., 2012).

Focused-Ion Beam Milling – CryoFIB - in a Dual Beam Scanning Electron FIB-SEM Cryo-Microscope

Owing to the lack of three-dimensional targeting methods (Fukuda et al., 2014), successful milling depends heavily on high-densities of the target structures. In nuclei of BK cells, required density of target structures had been confirmed by CEMOVIS sectioning. Vitreous samples on grids were mounted into modified Autogrids (FEI) to increase stability. These were transferred into a dual-beam FIB-SEM microscope (Quanta 3D FEG, FEI) by means of a cryo-transfer system (PP3000T, Quorum Technologies Ltd, Laughton, UK) and a custom-built transfer shuttle/sample holder (Rigort et al., 2010). During operation, samples were kept at a constant temperature below -180 °C using a homemade 360° rotatable cryo-stage (Rigort et al., 2012). To minimize curtaining artefacts during FIB milling, the sample was coated by organometallic platinum (GIS, FEI). Thin lamellae were prepared using Ga^{+} ions at 30 kV, under an effective beam to grid surface angle of 13°. Rough milling was performed with a rectangular pattern and 0.3 nA beam current, followed by sequentially lowered beam currents of 0.1 nA, 50 pA and 30 pA during the thinning and cleaning steps. Additionally, the specimen was observed by SEM at 5-10 kV and 5–50 pA.

Sub-Tomogram Averaging of CryoFIB/ET Data

The NEC units, forming a near continuous coat, did not provide clearly discernible features that might allow rational assignment of particle centres. Consequently, 31 vesicles from three tomograms were modelled as spheres to produce a series of 'particle' centres to be used for sub-tomogram averaging. The radius of each sphere was adjusted to the approximate mean radius of the NEC coat for each vesicle. Two out of three particle orientation parameters could be approximated using a 'particle axis' defined as normal to the sphere surface at each particle centre. Particle axes were then transformed to coincide with the Y-axis, and orientations around Y randomized to assist compensation of the 'missing wedge'. A spherically curved mask, 12 pixels (13.68 nm) thick, and 40 pixels wide was used to limit CCC to regions including protein signal, but not the vesicle membrane (VM). Tomograms were binned twice (to 11.4 Å/pixel), and initial averages calculated from a single vesicle of the highest quality and sphericalness over a cross-correlation box size of ~40 nm. This procedure involved a series of iterative CCC searches, in which translational search ranges were initially high, used a number of reference volumes selected from the vesicle itself as a reference template, and involved a full angular search around the particle axis (Y). For each starting reference, this approach resulted in averages showing pronounced hexagonal features with a centre-to-centre distance between unit cells (lattice spacing) of ~10 nm. Based on this observation, spherical sampling was optimized to oversample the NEC coat (nearest neighbour distance between points of ~3 nm). Sub-tomogram averaging was repeated for 9 vesicles after the initial result was centred on a 3-fold axis of symmetry and re-oriented to place the new particle axis on Y. To compensate for vesicle distortions that resulted in variable curvature of the NEC coat, a feature more pronounced for some vesicles than others, the CCC box was limited to a 26 pxl^3 box (~30 nm edge length), and an angular search range around Y of 120° was used. For each vesicle, cross-correlation searches were carried out over a number of iterations in which the angular range and step were made progressively finer, with final result being resubmitted for refinement using the same protocol until no improvements were observed. CCC was limited to a spatial frequency of 1/50 $Å^{-1}$ to ensure that alignment of noise did not significantly bias the alignment to the initial template. Duplicate particles (sharing particle centre coordinates) were removed after each iteration to produce an improved map for each vesicle from 200-350 particles. Final averages were calculated from 94 pxl^3 boxes and revealed that the NEC coat was in many cases ordered beyond the cross-correlated region. Additionally, the appearance of the VM, as well as other features in the map not involved in cross-correlation lent support to the validity of the alignment. Notably, while CCC searches were carried out about the 3-fold axes, the NEC averages exhibited 2-fold symmetry, and were consequently centred on the corresponding 2-fold axis, and the particle oriented around Y to place the *'6-2-6'* plane in coincidence with the X-axis. Owing to variability in curvature between vesicles – owing to size and shape - vesicle averages were not combined, nor was rotational symmetry applied to the vesicle maps at any stage. Resolution estimation was carried out for each vesicle

independently using the functions provided by PEET and did not follow the 'gold-standard' approach owing to the size of the vesicle particle datasets. Volumes were visualized in 3DMOD of IMOD (Kremer et al., 1996), or UCSF Chimera (Yang et al., 2012).

Construction of Soluble NEC Expression Vector

For expression of pU$_L$31 lacking the NLS (aa 26-271) and pU$_L$34 (aa 1-179) tagged with hexa-histidine (see Figure S3), the following primers were used to amplify the corresponding regions by PCR on genomic PrV strain Kaplan DNA as template:
U$_L$34-mpfc, CAC AGG ATC CGA CGA CCA TGA GCG GCA CC, BamHI, 31483-31502;
U$_L$34-mprc, CAC AAA GCT TGC GGG AGA CCG AGC GCT G, HindIII, 32027-32010;
U$_L$31-25F, CAC AGG TAC CGA TCG CTA CGC GCC CTA C, KpnI, 29507-29489 and
U$_L$31-mpr, CAC AGG TAC CCG GGC GAG GGG GGC GAA AG, KpnI, 28769-28787 with location numbers corresponding to Gene Bank Accession No. JQ809328.

Restriction enzyme sites were introduced for convenient cloning into corresponding restriction sites present in MCS1 (U$_L$34) and 2 (U$_L$31) of vector pETDuet-1 (Novagen). Correct amplification and insertion was verified by sequencing.

Small Angle X-Ray Scattering -SAXS - Data Collection, Processing, and Analysis

Data were collected from protein coming directly from a coupled size exclusion chromatography unit. The scattering patterns were measured in buffer B with 5 mM TCEP (tris(2-carboxyethyl)phosphine). The raw data were processed using PRIMUS (Konarev et al., 2003). The radii of gyration, R_g, of the particles were calculated from the Guinier approximation, as well as by use of the GNOM indirect transform package (Svergun, 1992), which calculated the distance distribution function *P*(r).

Ten independent *ab initio* models for the soluble NEC were computed with the simulated annealing *ab initio* bead modelling programs DAMMIF (Franke and Svergun, 2009). The ten models were aligned, averaged and filtered using the DAMAVER software suite (Volkov and Svergun, 2003). The model was further refined with DAMMIN (Svergun, 1999).

Architectural and SAXS-EM Integrative Model Building and Analysis

The sub-tomogram average is a 3.5-4-nm resolution representation of the NEC coat that does not unambiguously reveal the arrangement of heterodimers. The 'archway' and composing angular motifs are suggestive of a repeating feature that likely corresponded to this unit. Initially, we probed this assumption using a model of spheres corresponding to the approximate size of each component. Assuming a mass of ~30 kDa for both pU$_L$31 and pU$_L$34, each component of the NEC was initially modelled as a spherical volume ~3.5 nm in diameter. Guided by observation of lattice-type arrangements in the density, it was found that by placing these model spheres at the vertices of the lattices (Figure 4), a model could be devised which closely replicated the appearance of the experimental map for the NEC coat. The MD layer subunits were organized as clusters of 3 units (homotrimers). The placement of spheres reproduced and accounted for the 'angular' motif corresponding to a presumed heterodimer. The model subsequently served as the basis for the schematic shown in Figure 5. While this approach accounted for the 'pillars' of the 'archways', the nature of the 'arch' densities were initially difficult to reconcile, however the shared connection to the VM – also visible in raw tomograms - strongly suggests a homomeric interaction between pU$_L$34 in these regions with oligomerization taking place at sites distant (~3.2 nm) to the VM embedded C-terminal region (TM domain) – thereby forming the 'arch keystone'. Given the flexibility of the pU$_L$34 C-terminal part (Figure S3C), and that this is logically expected to be the closest part to the VM, the assertion was supported by our model. The quaternary model describes the hexagonal unit cell as consisting of a hexamer of pU$_L$31/34 heterodimers, and predicts at least three primary protein-protein interfaces contribute to assembly of the lattice in the first instance. These are the heterodimeric interface (pU$_L$31-pU$_L$34) which serves as the primary series of interfaces between the observed layers, as well as homomeric interactions of pU$_L$31 and pU$_L$34. Independently, pU$_L$31 was modelled initially

as a homotrimer, and these were placed on the vertices of the MD hexagonal lattice. It is equally possible that pU_L31 forms a homodimer in solution, with weaker interactions nucleating around the 3-fold axes resulting in coat formation. However, pU_L31 self-interaction in solution was not observed experimentally (Lorenz et al., 2015).

To investigate the orientation of soluble NEC within the cryoEM NEC coat unit cell – and move towards validation of the above model - we systematically cross-correlated $[pU_L31/34]_6$ SAXS models produced by varying the orientation of the heterodimer with respect to the cryoEM map. The SAXS PDB was converted to an EM density map (11.4 Å /pixel, 3 nm resolution) using the PDB2MRC program of the EMAN package (Tang et al., 2007). This map was centred on a 6-fold axis, oriented such that the NEC coat was perpendicular to Y-axis, and windowed to a 20 pxl^3 box. The SAXS map was thresholded tightly to produce an 'envelope' corresponding to a volume of ~87 nm^3. Thresholding served to approximately equalize internal densities such that these would not significantly influence the cross-correlation search against the cryoEM map (i.e. a fitting based on 'shape' alone was carried out). A series of hexameric models were created from $pU_L31/34$ envelopes using SPIDER by systematically varying six parameters (Shaikh et al., 2008). The first three parameters (phi, theta, psi) determined the orientation of the heterodimer within the unit cell, while translational parameters y, and x governed the displacement of the heterodimer from the NEC coat unit cell (particle average) centre along the Z- (unit cell radius) and Y- axis (vesicle radius), respectively. An angular step of 10° was used for the orientation search, while a step of 5.7 Å was used for the translation search. A hexameric model was created from the oriented and positioned envelope by populating heteordimers about the Y-axis according to 6-fold rotational symmetry. The final search parameter was a rotational search around the Y-axis, and was carried out over a 60° range (10° step) to identify each hexameric model best 'in-plane' orientation within the NEC coat unit cell. The SAXS-EM model supported interactions between components of the NEC coat proposed by the initial modelling (described above). Furthermore, the fit of the SAXS map allowed us to estimate the approximate number of amino acids contributing to each arch densities (C-terminal VM-connecting part of pU_L34 except the transmembrane domain) as: $pU_L34^{full\text{-}length}$-$pU_L34^{construct\text{-}length}$ (240-179) x 6 pU_L34 subunits/unit = 366 amino acids (~40.2 kDa). The sequence of pU_L34 is annotated in Figure S3C.

Modelling and Analysis of Curvature of the NEC Coat

Based on the observation of the two characteristic NEC layers (Figure 4), we hypothesized that the pattern of local interactions between components governs global properties of the vesicle by giving rise to 'well-defined' curvature when perpetuated over large surface areas; behaviour that should be amenable to modelling and analysis. The characteristic layers exhibited distinct hexagonal properties that could be represented by 'hypothetical lattices' for the MD and MP layers as shown in Figures 7A and S4. Serving as a convention for measurement, however, both layers correspond to a *p6* curved lattice. With respect to the integrative SAXS model, the MP and MD layers are positioned at either radial side of the heterodimer core ('pillar'); i.e. these coincide with locations where homomeric interactions are established and act to spatially constrain the placement of each subunit. Attempts to overlay the hypothetical lattices, i.e. as a geometric model of the NEC coat, showed these do not overlap precisely over distances larger than that of the unit of the MD lattice (Figure 7A). However, if we constrained the hexagonal unit centres of the shared unit cell to overlap (as would be carried out by multiple instances of macromolecular association), we find that precise curvature allows for radial alignment of the units (as shown in Figure 7B). The model suggested further that heteromeric association is required for curvature, and that these protein-protein interfaces are organized about the shared 2-fold axes of each layer (this was supported by SAXS-EM modelling, Figure 6). Notably, the geometric model predicts that it is this location of the heteromeric interactions that establishes the *'6-2-6'* directionality of curvature as observed in the experimental map (Figure 5 shows that the MD layer is – unexpectedly - planar between *'3-2-3'* axes), and consequently the modelling described below was carried out for the *'6-2-6'* cross-sectional plane.

To model curvature of the NEC coat, we derived an equation relating structural properties of the NEC to the radius to, or diameter between, the membrane distal layer(s) (MD) of the vesicle NEC coat (r_{MD} and d_{MD}, respectively). Equation 1 describes these in relation to the quotient ('*a*') of two arc lengths (V_{MP} and V_{MD}) between two adjacent 2-fold axes that are separated radially by a distance '*h*' (Figure 7).

$r_{MD}=h/(a\text{-}1)$, where $a=V_{MP}/V_{MD}$ (Equation 1)

The model assumes that the NEC coat achieves near complete coverage of the vesicles inner surface; an assumption supported by inspection of the tomograms. Conversely, use of the formula to successfully predict vesicle size based on local measurements from the NEC sub-tomogram average would serve to support that near complete coverage of the vesicle inner surface by the NEC coat does take place, and that the NEC coat architecture is perpetuated over most of this surface area. Sufficiently accurate measurement of these parameters should allow the appearance of the NEC coat to be modelled, as illustrated in Figure 7. *'h'* (~5.13 nm) is defined as the distance between distinct hexagonal arrangements of the MD and MP layers – coinciding with the 'pillars' of the archway, and '*a*' calculated as the quotient between arch lengths (as approximated from lattice spacing) between layers. The overall scheme for these measurements and respective calculations are described in Figure S4. To prepare for these measurements the NEC map was interpolated by a factor of two (5.7 Å/pixel), and tangential slices masked to include only well-defined unit cells, and subsequently padded by a factor of six to oversample the Fourier transform. r_{MD} (e.g. ~58.6 nm for the vesicle featured) can be measured from the experimental average in relation to the spherical model used for averaging, or as illustrated in Figures 7 and S4, calculated (predicted) from measurements taken locally from the experimental NEC coat average map. To scale arc lengths so as to construct the model overlay shown in Figure 7B, a radial projection of the NEC average was calculated using the BRADIAL program of BSOFT (Heymann et al., 2008), and this allowed the true magnitude of unit cell spacing (V_{MD}) to be measured. To produce the 2D *'6-2-6'* model overlay a hexagonal unit was created by modelling a heterodimer (the 'angular motif'), this was then displaced by half the measured V_{MD} with a reflection about the vertical axis to produce a unit cell cross-section. This unit model was then used to produce entire vesicle extrapolations by applying the angle subtended by the arc lengths measured from the sub-tomogram averages. This approach simulated the influence of the pU_L34 'arch', in spatially constraining the position of the heterodimer 'pillars'. According to this model the MD layer is thus formed by 2-fold and 3-fold interactions of pU_L31, while the MP layer varies with curvature, but is formed primarily by the 6-fold 'arch interactions', but also by interactions occurring on the 2-fold axes. The model predicts that complete coverage of the vesicle inner surface is geometrically allowed, as shown in Figure 7D, which is an extrapolation of that shown in Figure 7B.

SUPPLEMENTAL REFERENCES

Al-Amoudi, A., Studer, D., and Dubochet, J. (2005). Cutting artefacts and cutting process in vitreous sections for cryo-electron microscopy. J. Struct. Biol. *150*, 109-121.

Bleck, C.K.E., Merz, A., Gutierrez, M.G., Walther, P., Dubochet, J., Zuber, B., and Griffiths, G. (2010). Comparison of different methods for thin section EM analysis of *Mycobacterium smegmatis*. J. Microsc. *237*, 23-38.

Bouchet-Marquis, C., and Hoenger, A. (2011). Cryo-electron tomography on vitrified sections: a critical analysis of benefits and limitations for structural cell biology. Micron *42*, 152-162.

Dent, K.C., Hagen, C., and Grünewald, K. (2014). Critical step-by-step approaches toward correlative fluorescence/soft X-ray cryo-microscopy of adherent mammalian cells. In Correlative Light and Electron Microscopy II, T. Müller-Reichert, and P. Verkade, eds. (Burlington, MA: Academic Press), pp. 179-216.

Fukuda, Y., Schrod, N., Schaffer, M., Feng, L.R., Baumeister, W., and Lučić, V. (2014). Coordinate transformation based cryo-correlative methods for electron tomography and focused ion beam milling. Ultramicroscopy *143*, 15-23.

Gunkel, M., Schöneberg, J., Alkhaldi, W., Irsen, S., Noe, F., Kaupp, U.B., and Al-Amoudi, A. (2015). Higher-order architecture of rhodopsin in intact photoreceptors and its implication for phototransduction kinetics. Structure *23*, 628-638.

Hagen, C., Werner, S., Carregal-Romero, S., Malhas, A.N., Klupp, B.G., Guttmann, P., Rehbein, S., Henzler, K., Mettenleiter, T.C., Vaux, D.J*., et al.* (2014). Multimodal nanoparticles as alignment and correlation markers in fluorescence/soft X-ray cryo-microscopy/tomography of nucleoplasmic reticulum and apoptosis in mammalian cells. Ultramicroscopy *146*, 46-54.

Han, H.M., Zuber, B., and Dubochet, J. (2008). Compression and crevasses in vitreous sections under different cutting conditions. J. Microsc. *230*, 167-171.

Heymann, J.B., Cardone, G., Winkler, D.C., and Steven, A.C. (2008). Computational resources for cryo-electron tomography in Bsoft. J. Struct. Biol. *161*, 232-242.

Hsieh, C.E., Leith, A., Mannella, C.A., Frank, J., and Marko, M. (2006). Towards high-resolution three-dimensional imaging of native mammalian tissue: electron tomography of frozen-hydrated rat liver sections. J. Struct. Biol. *153*, 1-13.

Konarev, P.V., Volkov, V.V., Sokolova, A.V., Koch, M.H.J., and Svergun, D.I. (2003). PRIMUS: a Windows PC-based system for small-angle scattering data analysis. J. Appl. Crystallogr. *36*, 1277-1282.

Kremer, J.R., Mastronarde, D.N., and McIntosh, J.R. (1996). Computer visualization of three-dimensional image data using IMOD. J. Struct. Biol. *116*, 71-76.

Malhas, A., Goulbourne, C., and Vaux, D.J. (2011). The nucleoplasmic reticulum: form and function. Trends Cell Biol. *21*, 362-373.

Mastronarde, D.N. (2008). Correction for non-perpendicularity of beam and tilt axis in tomographic reconstructions with the IMOD package. J. Microsc. *230*, 212-217.

Mielanczyk, L., Matysiak, N., Michalski, M., Buldak, R., and Wojnicz, R. (2014). Closer to the native state. Critical evaluation of cryo-techniques for Transmission Electron Microscopy: preparation of biological samples. Folia Histochem. Cytobiol. *52*, 1-17.

Pierson, J., Ziese, U., Sani, M., and Peters, P.J. (2011). Exploring vitreous cryo-section-induced compression at the macromolecular level using electron cryo-tomography; 80S yeast ribosomes appear unaffected. J. Struct. Biol. *173*, 345-349.

Rigort, A., Bäuerlein, F.J.B., Leis, A., Gruska, M., Hoffmann, C., Laugks, T., Böhm, U., Eibauer, M., Gnaegi, H., Baumeister, W*., et al.* (2010). Micromachining tools and correlative approaches for cellular cryo-electron tomography. J. Struct. Biol. *172*, 169-179.

Schneider, G., Guttmann, P., Rehbein, S., Werner, S., and Follath, R. (2012). Cryo X-ray microscope with flat sample geometry for correlative fluorescence and nanoscale tomographic imaging. J. Struct. Biol. *177*, 212-223.

Shaikh, T.R., Gao, H.X., Baxter, W.T., Asturias, F.J., Boisset, N., Leith, A., and Frank, J. (2008). SPIDER image processing for single-particle reconstruction of biological macromolecules from electron micrographs. Nat. Protoc. *3*, 1941-1974.

Shao, L., Kner, P., Rego, E.H., and Gustafsson, M.G.L. (2011). Super-resolution 3D microscopy of live whole cells using structured illumination. Nat. Methods *8*, 1044-1046.

Sorzano, C.O.S., Messaoudi, C., Eibauer, M., Bilbao-Castro, J.R., Hegerl, R., Nickell, S., Marco, S., and Carazo, J.M. (2009). Marker-free image registration of electron tomography tilt-series. BMC Bioinformatics *10*, 124.

Svergun, D.I. (1992). Determination of the regularization parameter in indirect-transform methods using perceptual criteria. J. Appl. Crystallogr. *25*, 495-503.

Svergun, D.I. (1999). Restoring low resolution structure of biological macromolecules from solution scattering using simulated annealing. Biophys. J. *76*, 2879-2886.

Tang, G., Peng, L., Baldwin, P.R., Mann, D.S., Jiang, W., Rees, I., and Ludtke, S.J. (2007). EMAN2: An extensible image processing suite for electron microscopy. J. Struct. Biol. *157*, 38-46.

Turnbull, L., Strauss, M.P., Liew, A.T.F., Monahan, L.G., Whitchurch, C.B., and Harry, E.J. (2014). Super-resolution imaging of the cytokinetic Z ring in live bacteria using fast 3D-structured illumination microscopy (f3D-SIM). J. Vis. Exp. *91*, e51469.

Volkov, V.V., and Svergun, D.I. (2003). Uniqueness of *ab initio* shape determination in small-angle scattering. J. Appl. Crystallogr. *36*, 860-864.

Yang, Z., Lasker, K., Schneidman-Duhovny, D., Webb, B., Huang, C.C., Pettersen, E.F., Goddard, T.D., Meng, E.C., Sali, A., and Ferrin, T.E. (2012). UCSF Chimera, MODELLER, and IMP: an integrated modeling system. J. Struct. Biol. *179*, 269-278.
