# Supplementary figures and images for: Structural Basis of Vesicle Formation at the Inner Nuclear Membrane
Source: Cell. 2015 Dec 17;163(7):1692–701. doi: 10.1016/j.cell.2015.11.029 (PMC4701712; doi:10.1016/j.cell.2015.11.029)

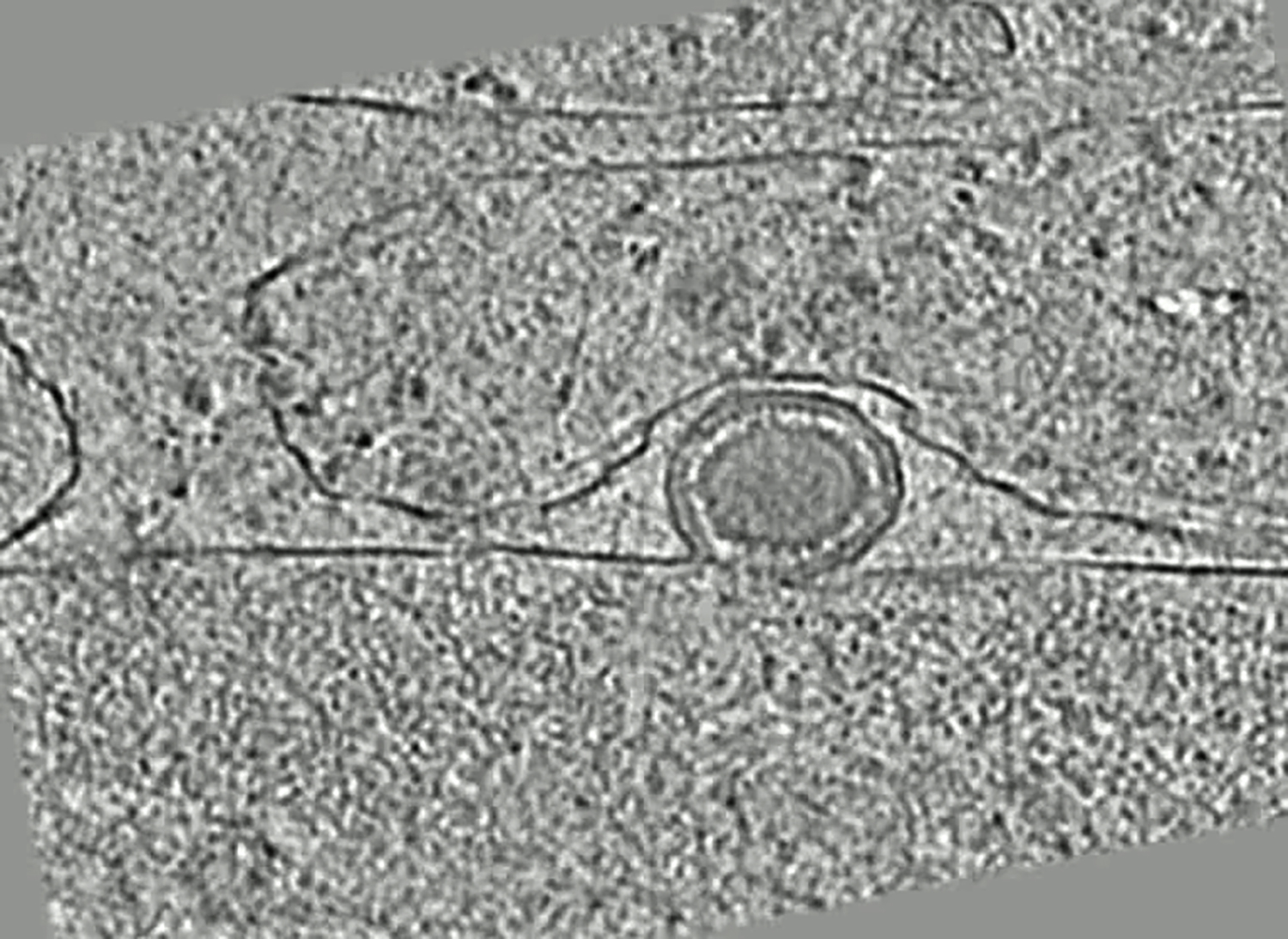

Supplement: Movie S1. Sliced View through Tomographic Reconstruction: CEMOVIS Data, Related to Figure 1D [file mmc2.jpg]

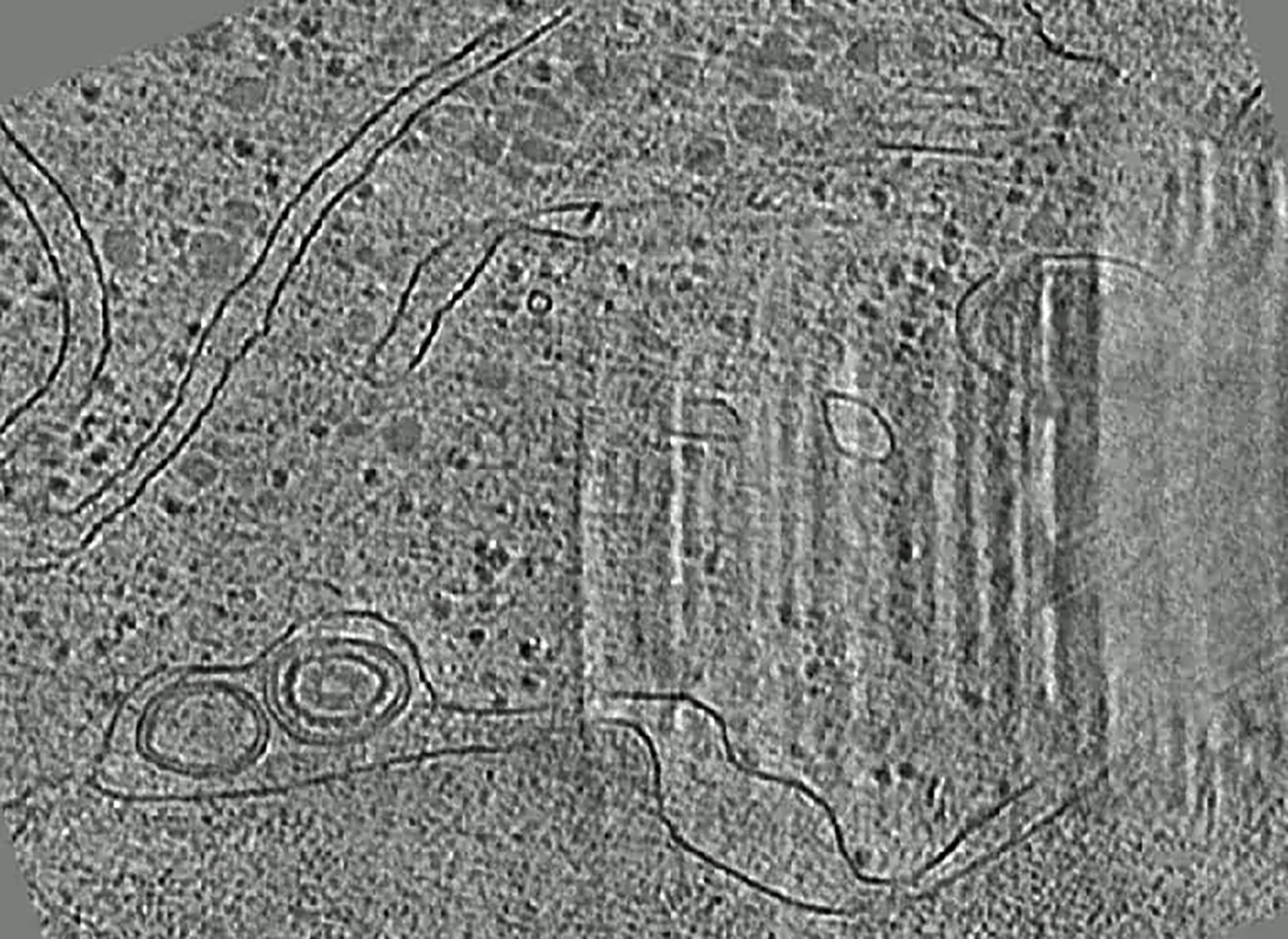

Supplement: Movie S2. Sliced View through Tomographic Reconstruction: CEMOVIS Data, Related to Figure 1E [file mmc3.jpg]

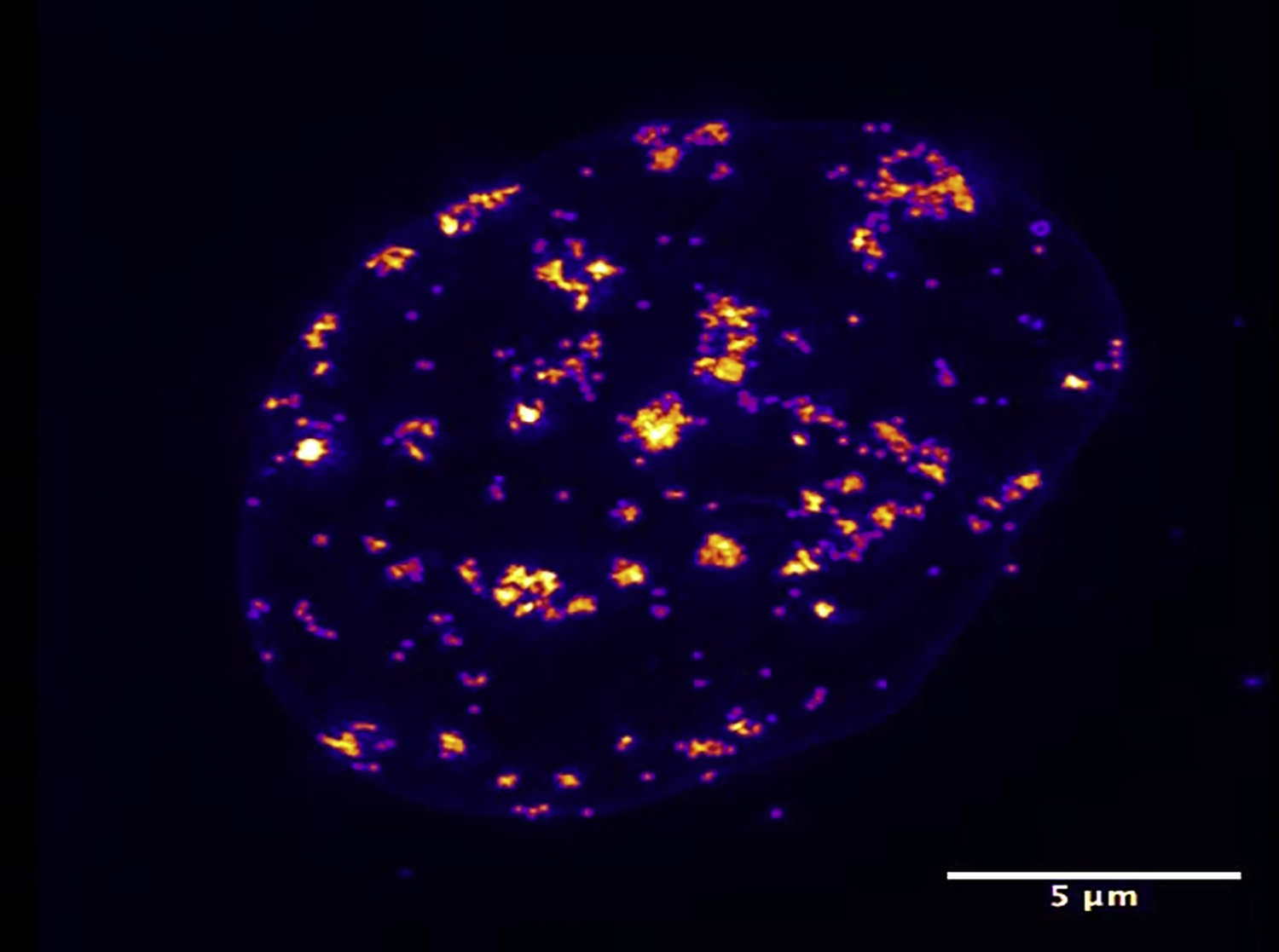

Supplement: Movie S3. Animated Volumetric Rendering of 3D-SIM Data, Related to Figure 2A [file mmc4.jpg]

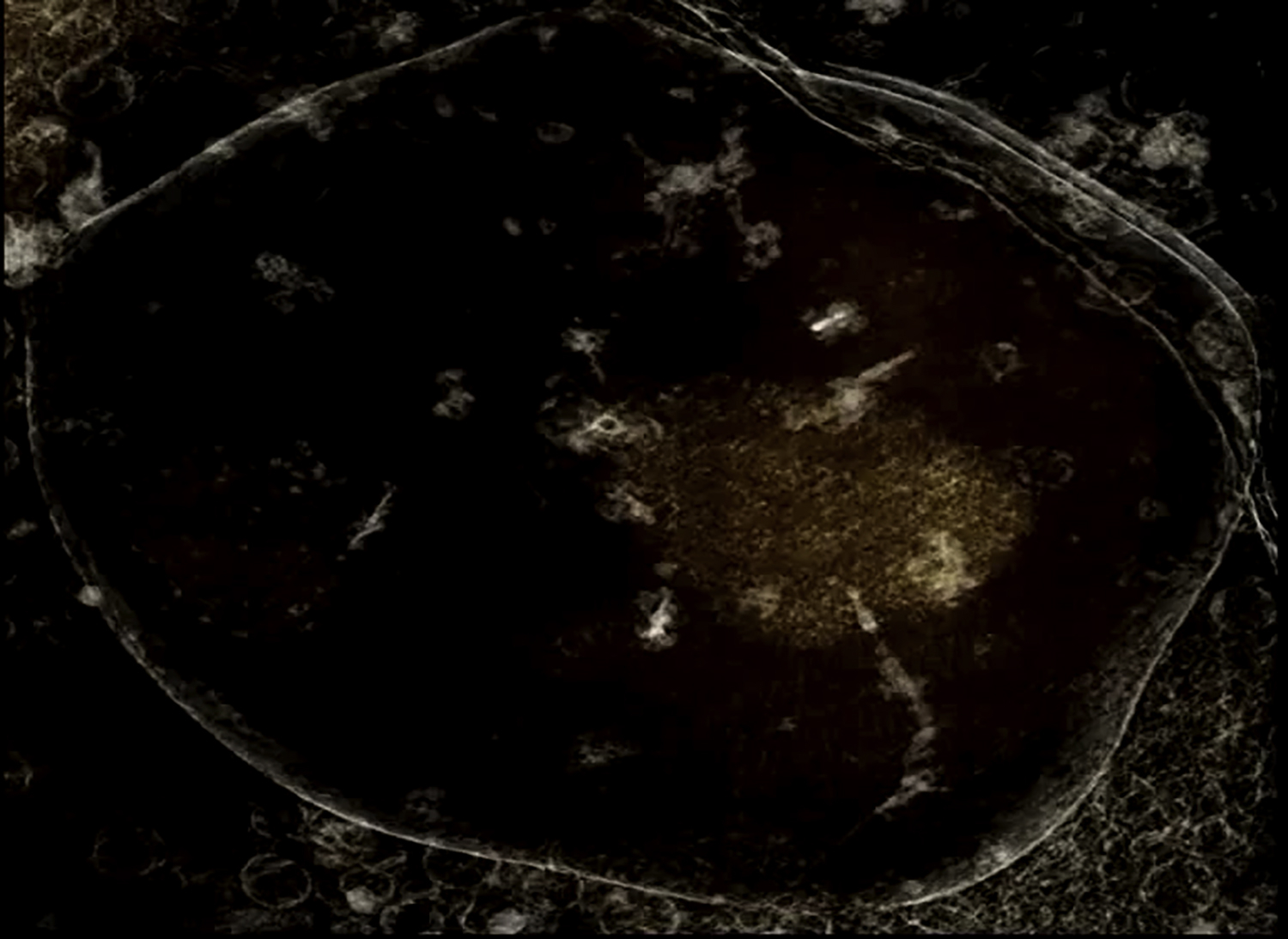

Supplement: Movie S4. Inverse Contrast Volume-Rendered CryoXT Reconstruction, Related to Figures S1D–S1D′′′ [file mmc5.jpg]

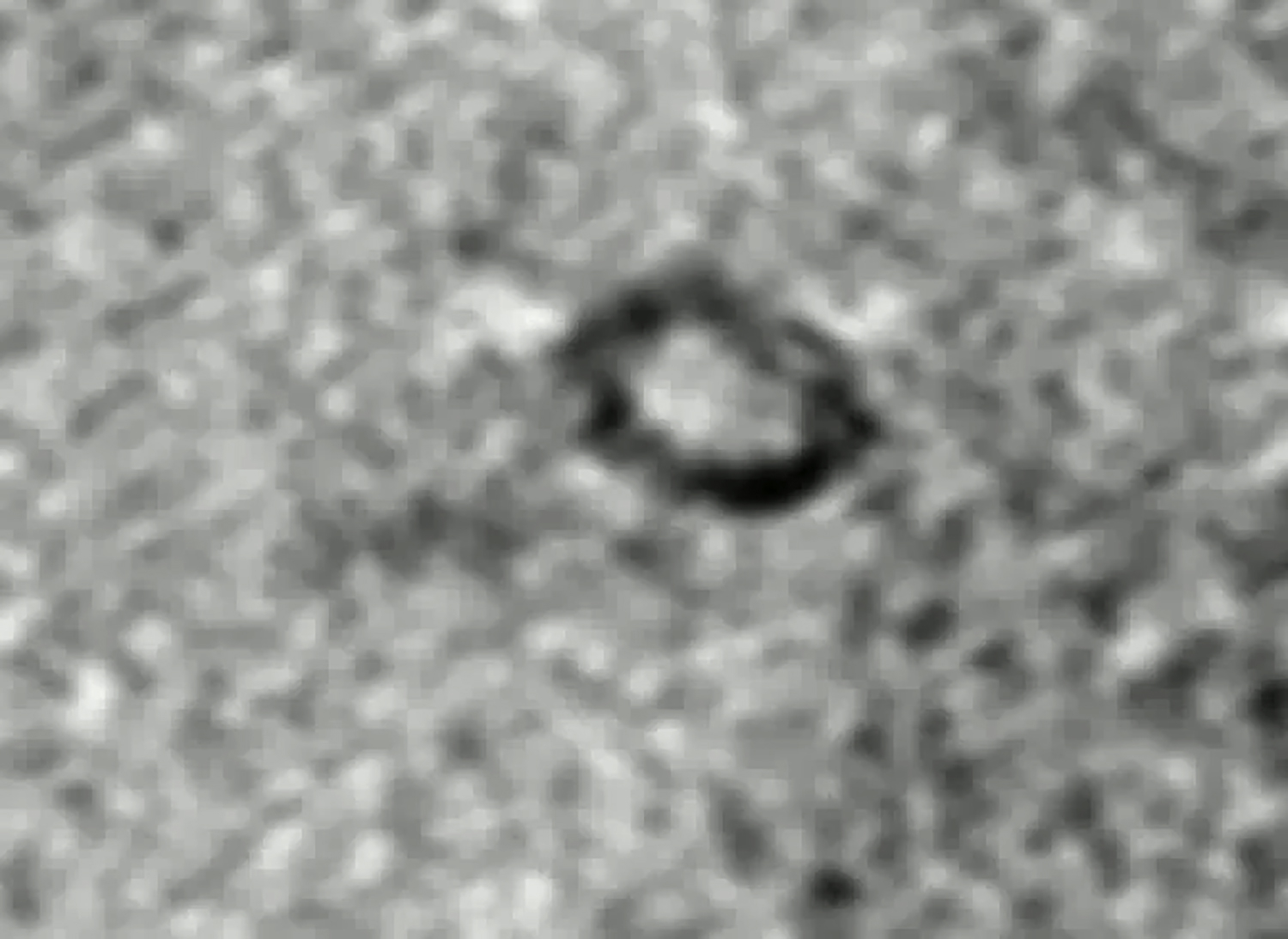

Supplement: Movie S5. Sliced View through the CryoXT Reconstruction Sub-volume and Isosurface Visualization, Related to Figure S1D′′′ [file mmc6.jpg]

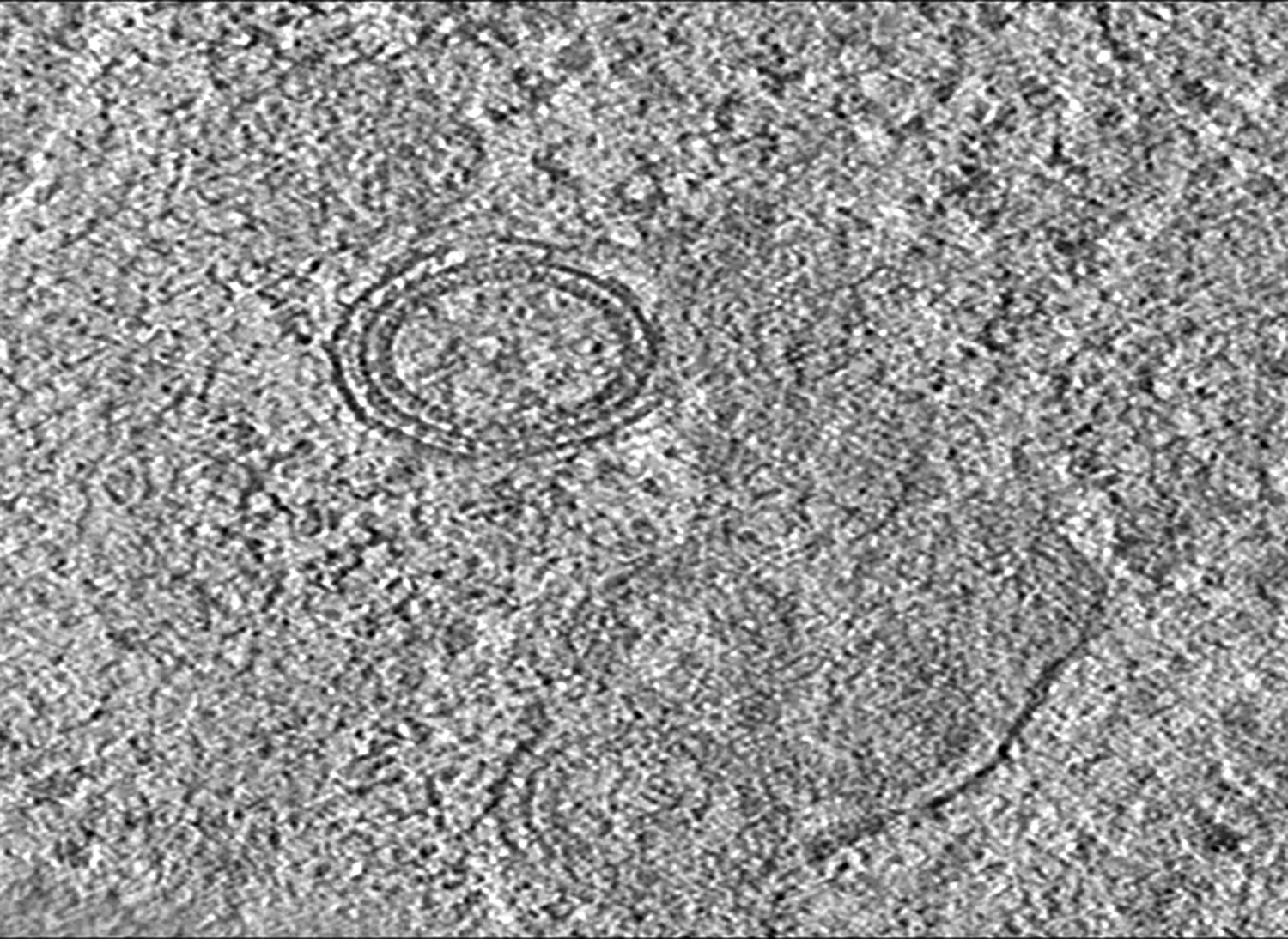

Supplement: Movie S6. Sliced View through the CryoFIB/ET Reconstruction, Related to Figure 3 [file mmc7.jpg]

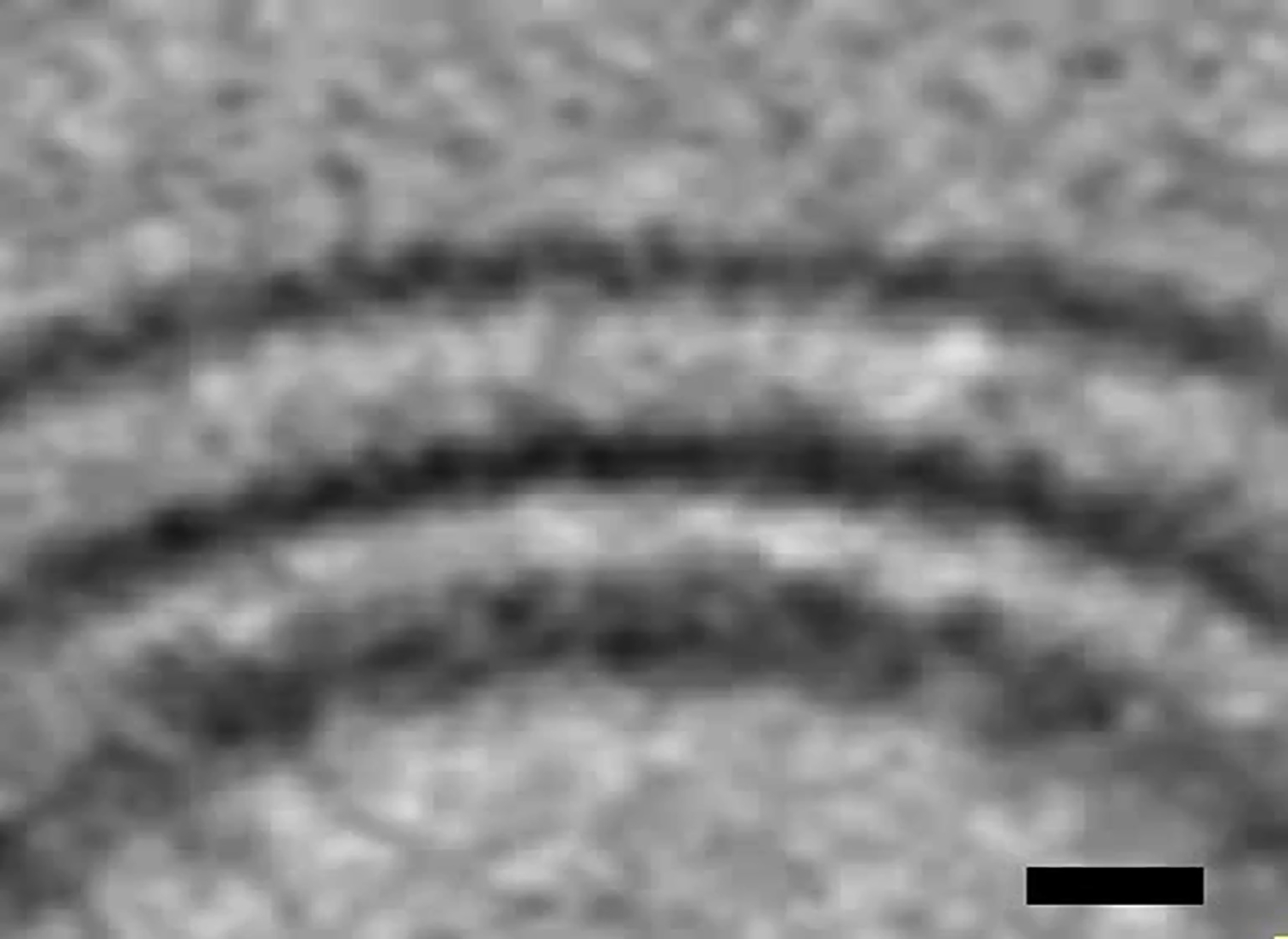

Supplement: Movie S7. XY Slices through the NEC Coat Sub-tomogram Average Parallel to “6-2-6” Cross-Section View, Related to Figures 4A, 5, 7B, and S4A [file mmc8.jpg]

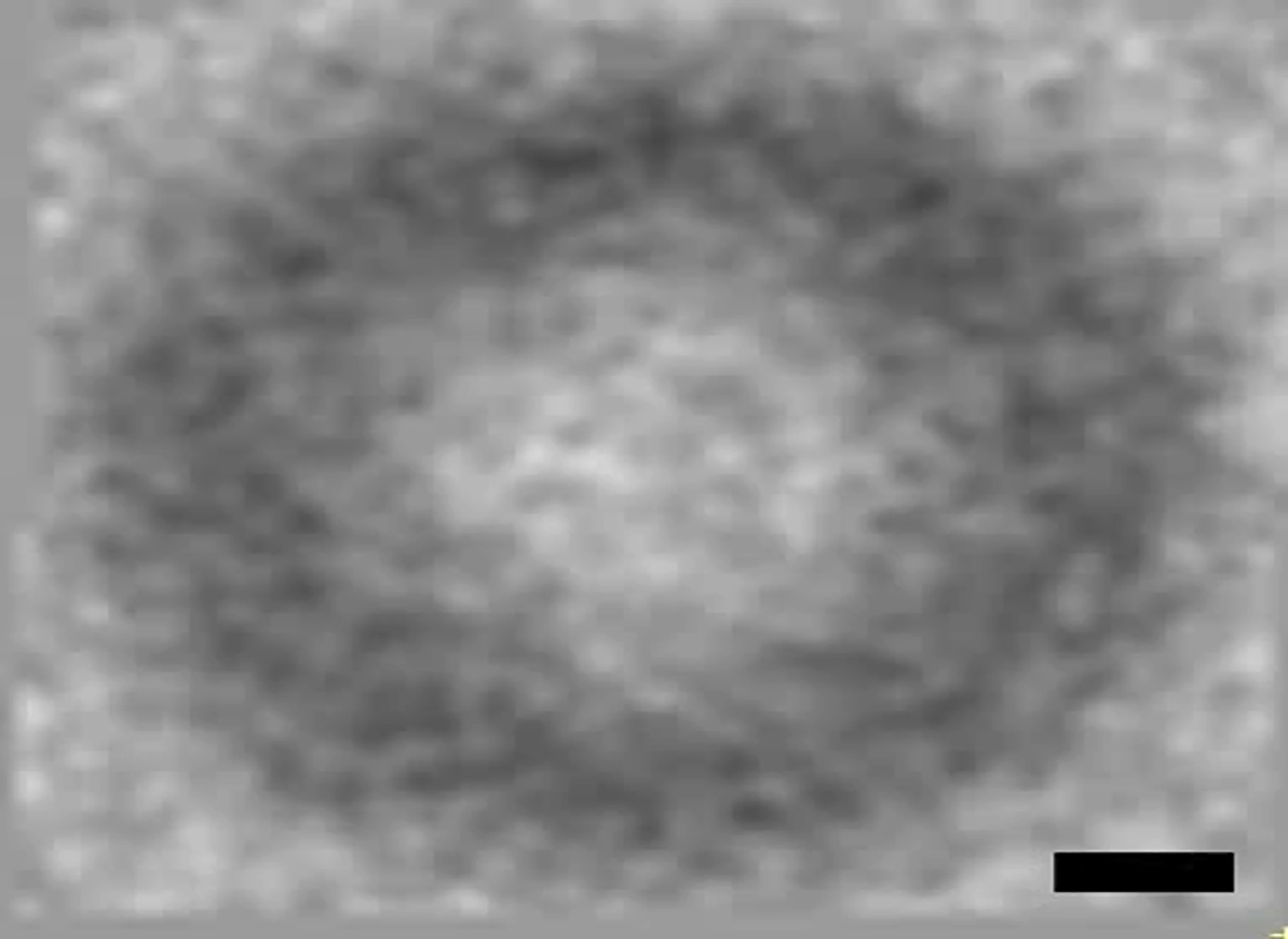

Supplement: Movie S8. Tangential XZ Slices through the NEC Coat Sub-tomogram Average, Related to Figures 4B and S4A [file mmc9.jpg]

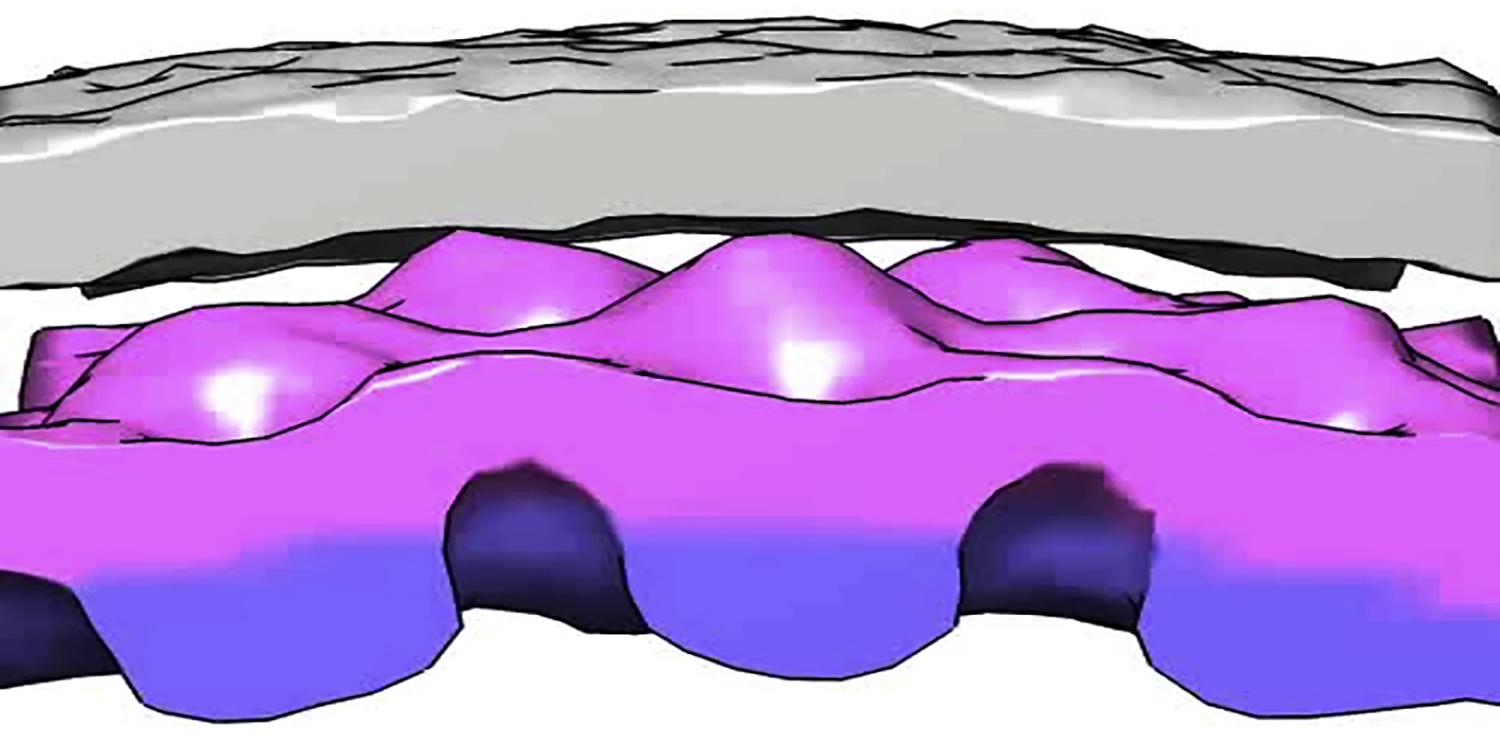

Supplement: Movie S9. Isosurface View of the NEC Coat Sub-tomogram Average, Related to Figure 4C [file mmc10.jpg]

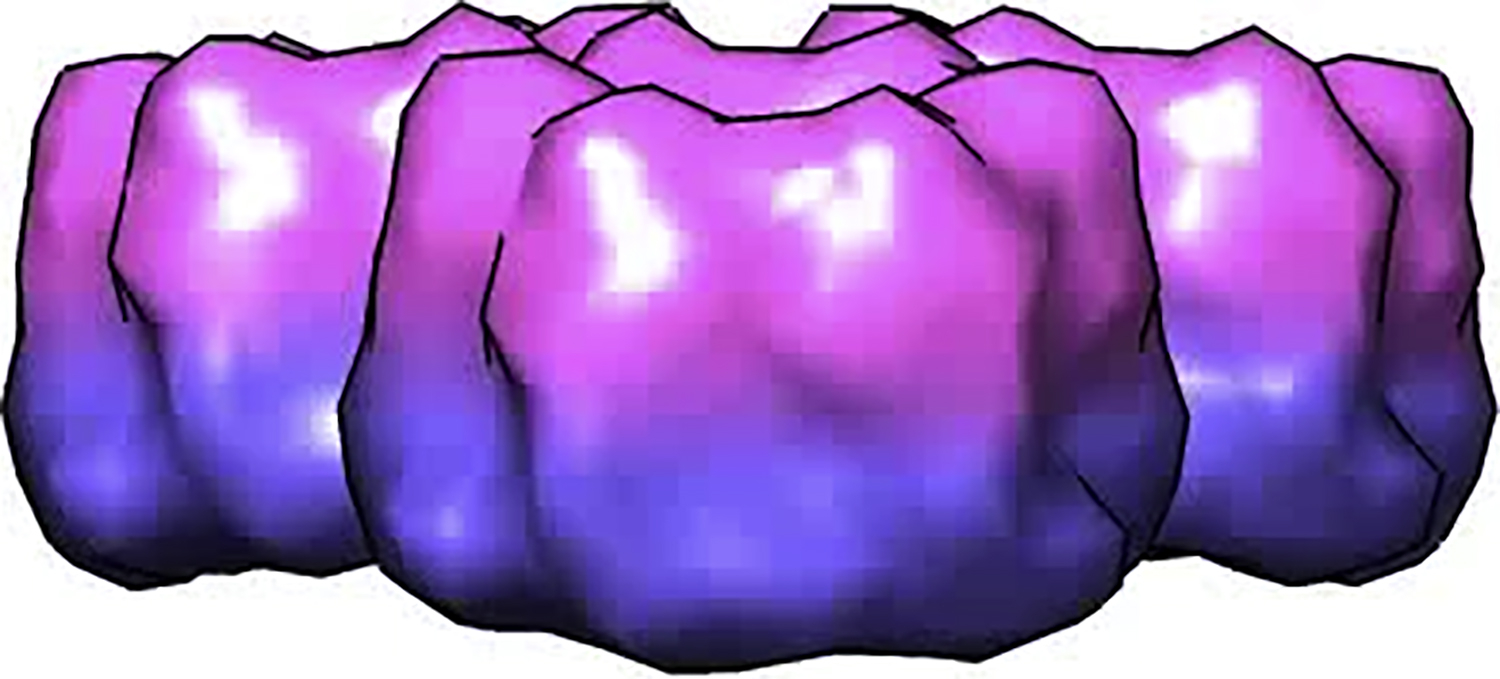

Supplement: Movie S10. Isosurface View of the NEC Coat Sub-tomogram Average, with Four Fitted SAXS-Derived Hexameric Models of the Heterodimeric NEC, Related to Figure 6 [file mmc11.jpg]
