# Supplementary material for: Structural Basis of Vesicle Formation at the Inner Nuclear Membrane
Source: Cell. 2015 Dec 17;163(7):1692–701. doi: 10.1016/j.cell.2015.11.029 (PMC4701712; doi:10.1016/j.cell.2015.11.029)
Supplement: Document S2. Article plus Supplemental Information [file mmc12.pdf]

Cell

Article

Structural Basis of Vesicle Formation at the Inner Nuclear Membrane

Graphical Abstract

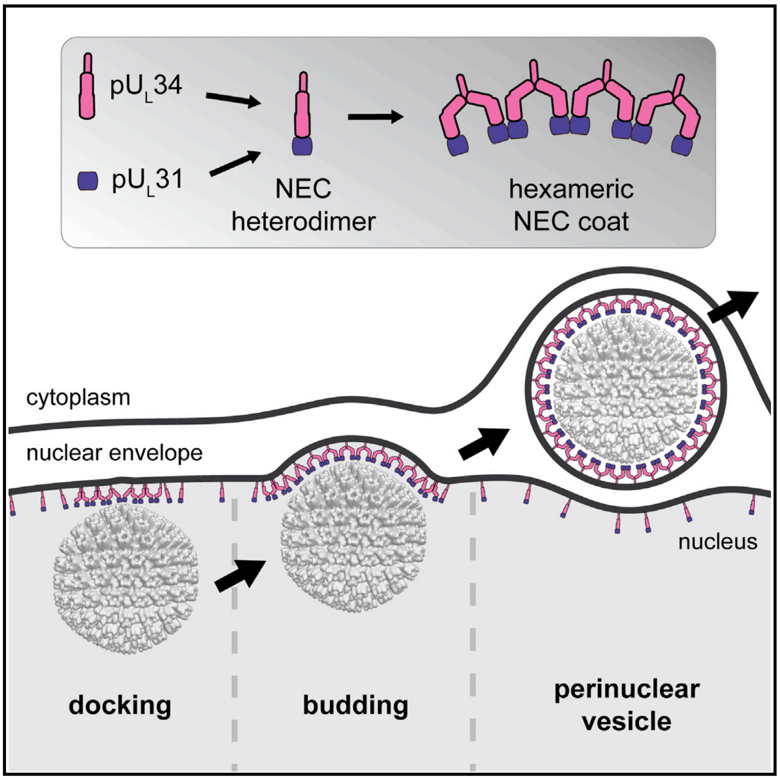

Highlights

- Multimodal imaging reveals mechanism of vesicle formation at inner nuclear membrane
- Nucleo-cytoplasmic cargo vesicle coat in situ comprises two distinct lattices
- Lattices are formed by hexameric building blocks made of the nuclear egress complex
- Induction of membrane curvature based solely on heterodimeric interactions

Authors

Christoph Hagen, Kyle C. Dent, Tzviya Zeev-Ben-Mordehai, ..., Jürgen M. Plitzko, Thomas C. Mettenleiter, Kay Grünewald

Correspondence

thomas.mettenleiter@fli.bund.de (T.C.M.),
kay@strubi.ox.ac.uk (K.G.)

In Brief

A multi-modal live-cell and cryo-imaging approach reveals how vesicles assemble at the inner nuclear membrane for transport to the cytoplasm during herpesvirus maturation. This also suggests a functional model for counterparts in uninfected cells that mediate nuclear egress of large cargo like ribonucleoprotein particles.

Hagen et al., 2015, Cell *163*, 1692–1701
December 17, 2015 ©2015 The Authors
http://dx.doi.org/10.1016/j.cell.2015.11.029

CellPress

Cell

Article

Structural Basis of Vesicle Formation at the Inner Nuclear Membrane

Christoph Hagen,[1,9] Kyle C. Dent,[1,2,9] Tzviya Zeev-Ben-Mordehai,[1,9] Michael Grange,[1,9] Jens B. Bosse,[3,9,10] Cathy Whittle,[1] Barbara G. Klupp,[4] C. Alistair Siebert,[1] Daven Vasishtan,[1] Felix J.B. Bäuerlein,[5] Juliana Cheleski,[1] Stephan Werner,[6] Peter Guttmann,[6] Stefan Rehbein,[6] Katja Henzler,[6,11] Justin Demmerle,[7] Barbara Adler,[8] Ulrich Koszinowski,[8] Lothar Schermelleh,[7] Gerd Schneider,[6] Lynn W. Enquist,[3] Jürgen M. Plitzko,[5] Thomas C. Mettenleiter,[4,*] and Kay Grünewald[1,*]

[1]Oxford Particle Imaging Centre, Division of Structural Biology, Wellcome Trust Centre for Human Genetics, University of Oxford, Roosevelt Drive, Oxford OX3 7BN, UK
[2]Diamond Light Source Ltd., Harwell Science and Innovation Campus, Didcot OX11 0DE, UK
[3]Department of Molecular Biology, Princeton Neuroscience Institute, Princeton University, Washington Road, Princeton, NJ 08544, USA
[4]Institute of Molecular Virology and Cell Biology, Friedrich-Loeffler-Institut, 17493 Greifswald-Insel Riems, Germany
[5]Department of Molecular Structural Biology, Max Planck Institute of Biochemistry, Am Klopferspitz 18, 82152 Martinsried, Germany
[6]Helmholtz Zentrum Berlin für Materialien und Energie GmbH, Wilhelm-Conrad-Röntgen Campus, 12489 Berlin, Germany
[7]Micron Oxford, Department of Biochemistry, University of Oxford, South Parks Road, Oxford OX1 3QU, UK
[8]Max von Pettenkofer-Institut, Ludwig-Maximilians-Universität München, Pettenkoferstr. 9a, 80336 Munich, Germany
[9]Co-first author
[10]Present address: Heinrich-Pette-Institute, Leibniz-Institute of Experimental Virology, Martinistrasse 52, 20251 Hamburg, Germany
[11]Present address: Paul Scherrer Institut, Swiss Light Source, 5232 Villigen PSI, Switzerland
*Correspondence: thomas.mettenleiter@fli.bund.de (T.C.M.), kay@strubi.ox.ac.uk (K.G.)
http://dx.doi.org/10.1016/j.cell.2015.11.029
This is an open access article under the CC BY license (http://creativecommons.org/licenses/by/4.0/).

SUMMARY

Vesicular nucleo-cytoplasmic transport is becoming recognized as a general cellular mechanism for translocation of large cargoes across the nuclear envelope. Cargo is recruited, enveloped at the inner nuclear membrane (INM), and delivered by membrane fusion at the outer nuclear membrane. To understand the structural underpinning for this trafficking, we investigated nuclear egress of progeny herpesvirus capsids where capsid envelopment is mediated by two viral proteins, forming the nuclear egress complex (NEC). Using a multi-modal imaging approach, we visualized the NEC in situ forming coated vesicles of defined size. Cellular electron cryo-tomography revealed a protein layer showing two distinct hexagonal lattices at its membrane-proximal and membrane-distant faces, respectively. NEC coat architecture was determined by combining this information with integrative modeling using small-angle X-ray scattering data. The molecular arrangement of the NEC establishes the basic mechanism for budding and scission of tailored vesicles at the INM.

INTRODUCTION

Intracytoplasmic transport between compartments is primarily mediated by vesicles (Schekman and Orci, 1996). These vesicles are shaped by specific coat proteins that are recruited to the site of assembly and function to deform the membrane (McMahon and Gallop, 2005). In contrast, movement into and out of the nucleus is effected by "gated transport" via the nuclear pore complexes (NPCs). NPCs allow free diffusion of small molecules and can mediate active transport of cargo up to ~39 nm in diameter (Panté and Kann, 2002). Larger macromolecular assemblies, however, are unable to pass through the NPC. Recently, vesicular trafficking was reported to mediate nucleo-cytoplasmic transport of ribonucleoprotein particles (Speese et al., 2012). This non-canonical pathway across the nuclear double membrane involves vesicle formation at the INM and fusion at the outer nuclear membrane (ONM). Another suggested role of this pathway is in nuclear recycling, i.e., transport of nuclear protein aggregates like defective NPC assembly intermediates to the cytosolic autophagy machinery for degradation (Rose and Schlieker, 2012; Webster et al., 2014).

The ribonucleoprotein particle transport mechanism is in many respects similar to nuclear egress of herpesviruses discovered a decade earlier (Mettenleiter et al., 2013). In the latter, ~125-nm-diameter icosahedral herpesvirus capsids assemble inside the nucleus and use vesicle-mediated transport across the nuclear envelope to gain access to the cytoplasm for further maturation. The combined evidence from the cellular ribonucleoprotein particle and viral capsid transport systems makes it likely that vesicle transport represents a general mechanism for translocation of large cargo from the nucleus to the cytoplasm that herpesviruses have usurped during evolution. Whereas the overall topology of the process of herpesvirus nuclear egress resembles cellular vesicle trafficking, little is known about the nanostructural details that lead to formation, scission, and fusion of INM-derived vesicles. Studied already in great biological detail, herpesvirus nuclear egress therefore represents a unique tractable model system

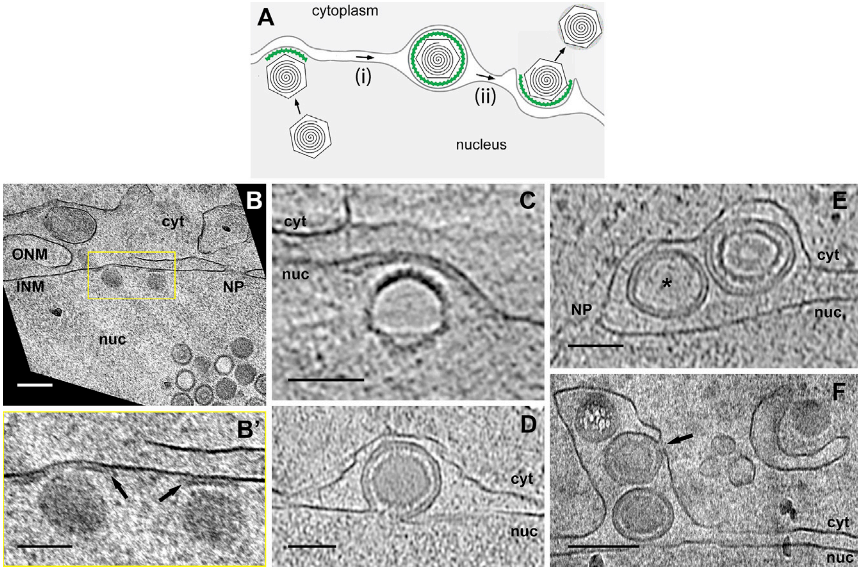

Figure 1. The NEC in the Replication Cycle of Herpesvirus

(A) Schematic of the stages of vesicle-mediated herpesvirus capsid nuclear egress, consisting of (i) primary envelopment by the NEC (green) at the INM and (ii) fusion of the vesicle with the ONM, resulting in de-envelopment to release the capsid into the cytoplasm.

(B–F) Developmental stages of the NEC coat in HSV-1-infected Vero cells (moi: 10, 16 hr p.i.) analyzed by electron cryo-microscopy of vitreous sections (CEMOVIS). (B) Projection image taken after pre-irradiation; nominal section feed: 30 nm; compression: 47%, corrected. (B′) Magnification of the yellow box marked in (B) (arrows: NEC coat). (C–E) Slices of tomographic reconstructions (C and D: nominal section feed, 100 nm; compression, 13%, 3D-corrected; E: nominal section feed, 50 nm; compression, 26%, 3D-corrected; asterisk, ILV; Movies S1 and S2). (F) Projection image taken after pre-irradiation; nominal section feed, 30 nm; compression, 47%, corrected. Scale bar, 200 nm (B and F) and 100 nm (B′–E). cyt, cytoplasm; INM, inner nuclear membrane; NP, nuclear pore; nuc, nucleus; ONM, outer nuclear membrane.

to delineate the general structural and functional basis of nucleo-cytoplasmic vesicle transport.

Studies of herpesvirus nuclear egress showed that, during infection, newly formed intranuclear capsids bud at the INM (Figure 1A) followed by membrane scission, resulting in enveloped capsids located in the perinuclear space. The envelope then fuses with the ONM to deliver the capsids to the cytoplasm. A large body of experimental studies has established that, throughout the *Herpesviridae*, two viral proteins, designated as pU_L31 and pU_L34 in the alphaherpesviruses herpes simplex virus 1 (HSV-1) and pseudorabies virus (PrV), form the heterodimeric nuclear egress complex (NEC; Figure 1A, green). The NEC is required and sufficient for vesicle formation, i.e., budding and scission, at the INM (Klupp et al., 2007; Mettenleiter et al., 2013). The C terminus of the type II membrane protein pU_L34 tethers the NEC to the INM, while pU_L31 is exposed to the nucleoplasm. pU_L31 then associates with the capsid surface in the lumen of the nascent perinuclear vesicle. After vesicle fusion with the ONM and release of the capsid, the NEC is exposed to the cytoplasm (Mettenleiter et al., 2013). NEC components are also likely to mediate cargo selection (Funk et al., 2015). Additionally, kinases recruited to the NEC are responsible for phosphorylation of lamins for local dissolution of the nuclear lamina to allow access of capsids to the INM (Hatch and Hetzer, 2014; Mettenleiter et al., 2013) and for phosphorylation of NEC components (Mou et al., 2009; Sharma et al., 2015). Whereas this prototypic budding process at the INM and its components are well characterized, the fusion process with the ONM is still under debate, including a possible role of viral fusogenic glycoproteins (Mettenleiter et al., 2013).

Recently, in vitro studies showed that partially truncated NEC components artificially membrane tethered to giant unilamellar vesicles formed a coat that can function as a minimal virus-encoded vesiculation machinery, not requiring additional viral or cellular factors (Bigalke et al., 2014). Furthermore, artificial membrane tethering of pU_L31 alone was sufficient for induction of membrane invaginations and membrane scission in giant unilamellar vesicles (Lorenz et al., 2015). However, owing in part to the reduced complexity of the models used, these studies did not provide sufficient ultrastructural detail to elucidate the architecture and functionality of the NEC coat. Thus, we here investigated the NEC in its native location, in vesicles at the periphery of the nucleus.

The size of the nucleus makes it a challenging target for visualization of intra-nuclear structures at molecular resolution in situ. Nevertheless, by applying an integrated multi-modal approach that enabled near-native imaging over variable scales and resolutions (Zeev-Ben-Mordehai et al., 2014), we were able to characterize in detail both the extent of nuclear membrane remodeling and the architecture of the NEC at the INM. We first show in cryo-sections of herpesvirus-infected cells that the NEC forms a protein coat that lines capsid-containing perinuclear vesicles during egress. We then characterize the ultrastructure of NEC-coated vesicles in non-infected cells that co-express pU_L31 and pU_L34. Further, that latter experimental system provided a higher frequency of these vesicles, allowing successful cellular electron cryo-tomography of lamellae prepared by advanced focused ion beam cryo-milling (cryoFIB). Subsequent three-dimensional averaging of the NEC coat revealed that it forms an ordered lattice with two different hexameric faces. X-ray scattering data of solubilized NEC complexes, combined with integrated modeling, allowed us to determine that these two faces represent pU_L34 anchored in the vesicle membrane and pU_L31 forming the inner layer. The unique structure and interactions between the two protein layers result in a defined membrane curvature, ensuring that viral capsids are tightly enveloped. Our data reveal how formation of correctly sized perinuclear vesicles is achieved and establish a mechanistic basis for nucleo-cytoplasmic transport of large cargoes.

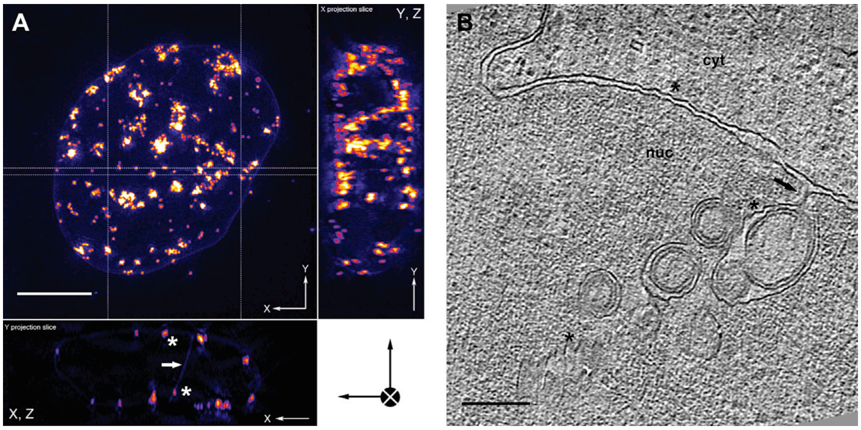

Figure 2. Nuclear Ultrastructure in PrV pU$_{L}$31/pU$_{L}$34-GFP Co-Expressing Cells

(A) Slices through a 3D volume of a porcine epithelial-like embryonic kidney cell determined by live-cell 3D-SIM at 37°C (~120 nm lateral resolution). The cell nucleus, as well as features of the nuclear envelope (arrow, tubular invagination; asterisks, vesicle clusters or "speckles"), are highlighted by stably co-expressing PrV pU$_{L}$31 and pU$_{L}$34, the latter fused to GFP. Thickness of XZ and YZ projections is indicated by dashed lines in the XY projection (for 3D volume, see Movie S3). Scale bar, 5 μm.

(B) A slice of a CEMOVIS tomographic reconstruction of the nuclear periphery of a proteinase-K-detached BK cell cryo-immobilized after 2 days standard cultivation (nominal section feed, 100 nm; compression, 13%, 3D-corrected) depicts the typical size range of ILVs in an invagination of the INM (arrow, "stalk" region; asterisks, membrane crevasses). Scale bar, 200 nm. cyt, cytoplasm; nuc, nucleus.

RESULTS AND DISCUSSION

In Situ Structural Characterization of NEC-Mediated Capsid Envelopment at the INM by CEMOVIS

To analyze the NEC coat formed in situ during viral infection in its most native environment, we used electron cryo-microscopy and tomography (cryoEM/T) (Hoenger, 2014). CryoET imaging of areas deeper inside cells typically requires vitreous sections in order to provide electron transparent specimens of <500 nm thickness (Lučić et al., 2005). In electron cryo-microscopy of vitreous sections (CEMOVIS), a method for imaging hydrated and unstained cellular ultrastructural detail (Dubochet, 2012), NECs were observed as electron-dense coats at the nucleoplasmic side of the INM and in the perinuclear space of HSV-1-infected Vero cells (Figures 1B–1F and Movies S1 and S2). When nuclear capsids were in close contact to the INM, a planar NEC coat of ~100 nm diameter, i.e., about the width of the capsid, was observed (Figure 1B′, right HSV-1 capsid). The coat curved and expanded during budding of the INM into the perinuclear space (Figure 1B′, left HSV-1 capsid). Interestingly, the electron-dense NEC coat did not extend beyond the individual sites of budding (Figures 1C and 1D and Movie S1). Ultimately, the NEC formed a tightly fitting complete coat around the capsid (Figures 1D–1F and Movies S1 and S2). In HSV-1-infected Vero cells, not only DNA-filled C-capsids underwent primary envelopment (Figures 1B, 1D, and 1F), but also empty A-capsids (Figure 1C) and scaffold-containing immature B-capsids (Figure 1E, right vesicle). Intraluminal vesicles (ILVs, defined as possessing the NEC coat but lacking capsids; Figure 1E, asterisk; Movie S2) represented 34% of all observed perinuclear vesicles in HSV-1-infected Vero cells (14 of 41 vesicles, from 21 tomograms total), with a mean inner diameter of 115 nm ± 11 nm SD (n = 12). During de-envelopment, the NEC coat was left behind at the cytoplasmic face of the outer nuclear membrane (Figure 1F, arrow), and cytoplasmic capsids, now devoid of the NEC coat, subsequently underwent virion assembly (Figure 1F, right, and Movie S2, right upper-corner). This result contradicts previous conclusions drawn on the basis of interpreting densities in heavy-metal-stained, freeze-substituted, plastic-embedded samples (Wild et al., 2015) and is in line with the absence of pU$_{L}$31 and pU$_{L}$34 in extracellular HSV-1 virions (Loret et al., 2008).

A grainy nature of the NEC coat was readily visible in computational slices through cryoET reconstructions (Figures 1C–1E and Movies S1 and S2), suggesting a modular lattice-type architecture consisting of repetitive units. To analyze the structure and function of this coat in greater detail, a multimodal imaging approach was needed, spanning several length scales and covering from the nuclear distribution of its fully assembled form down to interactions of its single constituents.

Nuclear Ultrastructure in an In Situ Cell Model for Elucidating the NEC Architecture

In HSV-1-infected cells, the number of capsid envelopment events captured at the INM was low. Therefore, we used a previously described porcine cell line that stably co-expresses pU$_{L}$31 and pU$_{L}$34 of PrV as a model frequently showing NEC-mediated vesicle formation (Klupp et al., 2007). In this BK cell line, pU$_{L}$34 is anchored to the INM by its authentic C-terminal transmembrane region, with the C-terminal GFP tag exposed on the vesicle outside, i.e., on the opposite membrane side of the NEC. Tagging allowed visualization of the NEC in vivo. By using three-dimensional structured illumination microscopy (3D-SIM) (Schermelleh et al., 2010), volumetric live-cell imaging of the nucleus at sub-diffraction resolution was achieved. This revealed clusters of fluorescent speckles of ~160–1,500 nm diameter at multiple sites around the nuclear periphery, as well as within the nuclear interior along membranous invaginations (Figures 2A and Movie S3). These clusters represent accumulations of NEC-containing vesicles in the perinuclear space (Klupp et al., 2007) and were intensely fluorescent, suggesting high local concentrations of pU$_{L}$34-GFP.

Imaging of similar regions of BK cells at higher resolution by soft X-ray cryo-microscopy/tomography, guided by correlation with GFP fluorescence (Hagen et al., 2012), provided detailed information about the spatial distribution of the NEC-containing target structures/vesicle clusters throughout the nucleus (Figure S1 and Movies S4 and S5). The ultrastructure of these

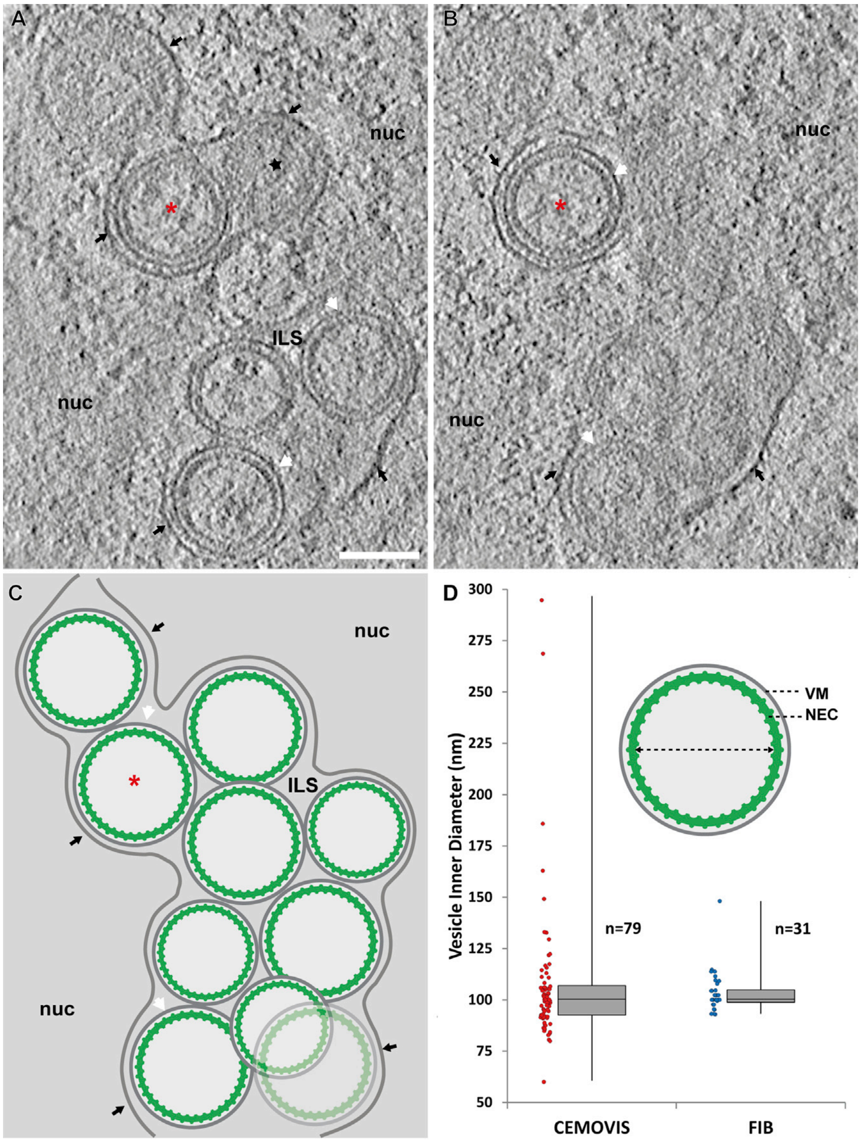

Figure 3. Ultrastructural Characterization of a Cluster of ILVs by CryoFIB/ET

(A–C) Slices through an electron cryo-tomogram of a lamella prepared by cryoFIB, interpreted schematically in (C). Vesicles not shown in the experimental map slices are depicted semi-transparently (Movie S6). ILVs are tightly surrounded by the INM (black arrows) and are closely related in size, exhibiting diffuse contents. The NEC coat (green) appears to nearly cover the entire inner surface of the vesicle membrane (white arrows). A fuzzy layer of density attributed, at least partly, to the C-terminal GFP of the type II transmembrane protein construct pU_L34-GFP, surrounds each vesicle and projects into the intraluminal/perinuclear space (ILS). Inspection of tangential slices (example: black star) suggests that imperfections in the lattice arrangement of the NEC coat do occur but that these likely represent only a small fraction of the total vesicle surface area. Red asterisks highlight a near spherical vesicle from which measurements of NEC coat parameters were taken. Scale bar, 100 nm. nuc, nucleus.

(D) Boxplot of the distribution of vesicle sizes (vesicle inner diameter: dashed line in inset vesicle) measured in 3D from BK cells prepared for tomographic electron imaging by CEMOVIS (red) and cryoFIB (blue). The distributions share a median of ~100 nm. VM, vesicle membrane.

intranuclear vesicle clusters was next characterized in 3D by CEMOVIS (Figure 2B), resulting in visualization of vesicles with a grainy inner NEC coat and a mean inner diameter of 107 ± 33 nm SD (n = 79). These vesicles were closely similar in size and structure to the capsid-less ILVs in infected cells (Figure 1E, asterisk).

Characterization of larger volumes by serial CEMOVIS sections enabled us to localize and characterize the occurrence of repetitive NEC structures/lattices suited for sub-tomogram averaging, even in rare developmental states (Figure 1). An alternative cryo-thinning technique, cryoFIB, has recently been developed to produce 100–300 nm thick lamellae from vitreous samples. This approach does not rely on physical cutting and, thereby, avoids sectioning artifacts (Marko et al., 2007; Rigort et al., 2012). CryoET data were recorded from cryoFIB-prepared lamellae of plunge-frozen BK cells. Perinuclear vesicles were typically spherical, although some exhibited a more irregular shape (Figures 3A–3C and Movie S6), possibly due to crowding. The NEC protein layer was evident as a clear lattice-like, ~10-nm-thick coat lining the entire inside of each vesicle with periodic connections to the vesicle membrane (Figures 3A and 3B). Size measurements of vesicles from three tomograms showed a peaked distribution with a mean inner diameter of 103 ± 10 nm SD (n = 31) (Figure 3D). The thickness of the coat and the size of ILVs in BK cells measured in 3D from CEMOVIS and cryoFIB-based data were in agreement (Figures 2B and 3 and Movie S6) and were similar to CEMOVIS data from HSV-1-infected Vero cells (Figure 1E). The vesicle diameters did not show a Gaussian distribution. Instead, the distribution is heavily skewed and peaked with very light tails (Figure 3D). These properties suggest that a specific mechanism inherent to NEC coat assembly is a predominant determinant of vesicle size, with positive skewness indicating a lower limit of the measured parameter.

Ultrastructure of the NEC Coat Lattice Revealed by Sub-Tomogram Averaging

The previous observations suggested a highly repetitive organization of the NEC protein layer. Taking different curvature into account, the structure of the NEC coat was therefore determined independently for each vesicle by sub-tomogram averaging from the cryoFIB/ET data (Figure 4). Each vesicle average revealed a curved hexagonal lattice composed of two

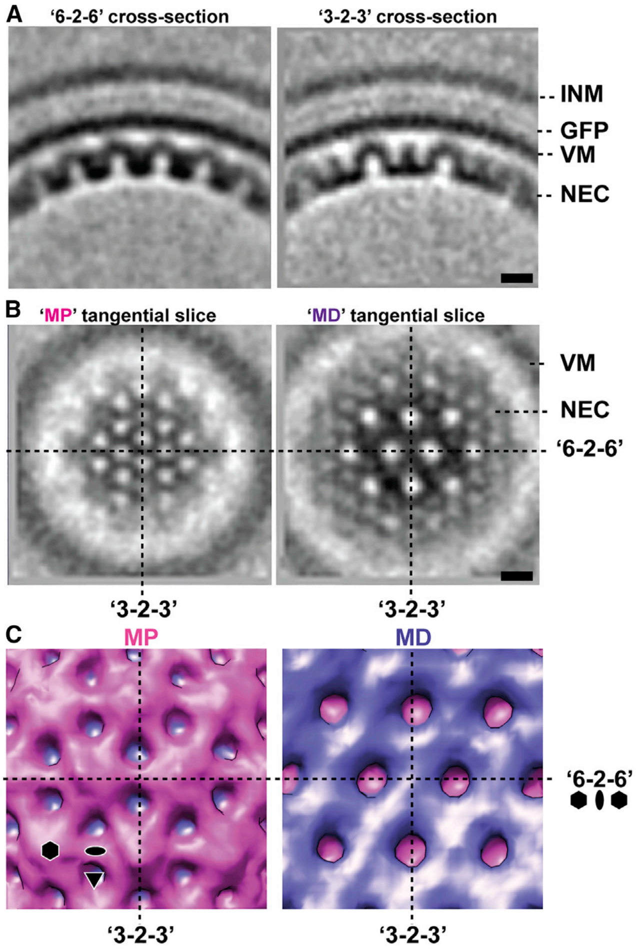

Figure 4. Sub-tomogram Averaging of the NEC Coat

(A) Sub-tomogram average (~3.5 nm resolution, Figure S2) viewed in characteristic "*6-2-6*" and "*3-2-3*" cross-section, oriented such that the slice passes through a 2-fold axis and intercepts adjacent 6-fold (Movie S7) or 3-fold axes, respectively. Scale bar, 10 nm. VM, vesicle membrane.

(B) Tangential slices through membrane proximal (MP) and membrane-distal (MD) layers (Movie S8). Each layer corresponds to *p6* symmetry with lattice spacing of ~11 nm. However, the MP layer exhibits a distinct arrangement of density that, at the available resolution, appears to correspond to a *p6* lattice with a spacing of ~6 nm and offset from the MD lattice by 30°. Scale bar, 10 nm.

(C) Surface views rendered from the vesicle exterior and the vesicle interior show characteristic features of the MP and MD layers, respectively (Movie S9). Symbols indicate symmetry axes (6-fold, 3-fold, and 2-fold).

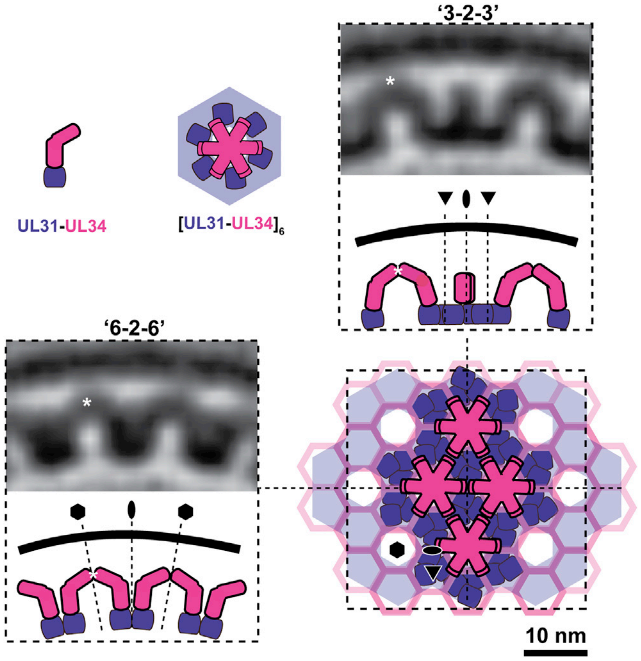

Figure 5. Interpretation of the NEC Coat Based on the Sub-tomogram Average

The initial architectural model of the NEC coat (lower-right) made from hexameric interactions of $pU_L31/34$ heterodimers (hexameric unit cell; upper-middle) is shown alongside characteristic cross-sectional views of the NEC coat average ("*6-2-6*" and "*3-2-3*" views shown in Figure 4A and "*6-2-6*" in Movie S7). An arch keystone is marked with a white asterisk. pU_L34 (magenta) and pU_L31 (purple) make up the MP and MD layers, respectively.

tightly interconnected layers of distinct appearance (Figures 4A, 4B, and S2 and Movies S7 and S8 [resolution 3.5–4 nm]). We termed the two NEC layers the membrane-proximal (MP) and membrane-distal (MD) layers. The MD layer is a ~3-nm-thick hexagonal lattice with a spacing between repeating unit centers of ~11.5 nm (purple), and the MP layer is ~7 nm thick (magenta) (Figure 4C and Movie S9). In cross-sections, the repeating unit of the NEC coat (a single hexagon) shared a characteristic "archway" motif—similar to an inverted 'U' (Figure 4A and Movie S7). The hexagonal unit could be further decomposed into a motif of angular appearance, one side of the archway, which appeared kinked at approximately two-thirds along its length. Thus, the MP layer consists of a conical arrangement of six independent densities that originate at the unit cell "keystone" extending toward the vesicle center to form an "arch" and connecting with the MD layer near the 2-fold axes. Densities connecting the arch/keystone of the unit cell to the vesicle membrane were already apparent in raw tomograms (Figure 3 and Movie S6) and became accentuated after sub-tomogram averaging (Figure 4A and Movie S7).

We assigned the MP layer to the membrane-anchored pU_L34 and the MD layer to pU_L31 (Figure 4C and Movie S9). The schematic interpretation shown in Figure 5 is based on analysis of tangential slices revealing the characteristic arrangements of protein density in each layer (Figure 4B and Movie S8). Orthogonal cross-section slices, shown adjacent to the model (Figure 5), reveal that the local curvature of the MD layer (pU_L31) is not isotropic. Between 3-fold axes ("*3-2-3*") the MD layer is distinctly planar, whereas between 6-fold axes ("*6-2-6*"), the curvature of MD is consistent with that of the vesicle membrane.

Integrative Modeling of the NEC Lattice Structure Using a Small-Angle X-Ray Scattering Envelope for the Soluble $pU_L31/34$ Heterodimer

The cryoFIB/ET sub-tomogram averages do not readily reveal the stoichiometry of $pU_L31/34$ heterodimers in the NEC coat. The schematic interpretation shown in Figure 5 suggests that

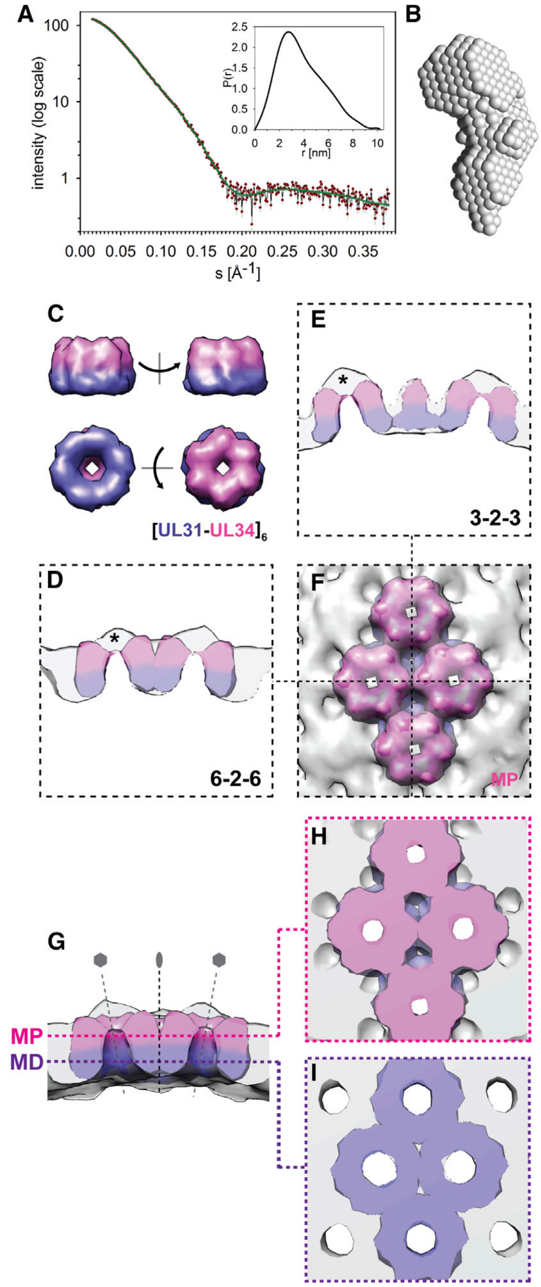

Figure 6. Stoichiometry of the $pU_L31/34$ Heterodimer within the NEC Coat, Based on SAXS Data

(A–F) (A) SAXS scattering curve for soluble PrV NEC (red circles; Figure S3) with the fit of the theoretical scattering curve calculated from the ab initio model (green line) and the respective size distribution (inset). (B) The ab initio heterodimeric model derived from the simulated annealing bead modeling of the 1D-SAXS curve. (C) Surface views of the SAXS-based hexameric model ($[pU_L31/34]_6$) accounting best for the cryoEM-derived density. One hexamer is rotated to show four views from different directions. (D–F) Four copies of $[pU_L31/34]_6$ fitted into the cryoEM map (transparent gray surface, compare Movie S10) are shown in both cross sections (D and E) as well as semi-transparent surface view from the vesicle exterior (F). Note the missing density at the center of each hexamer (the arch keystone, instances marked with a black asterisk in D and E), as the model is based on a SAXS model of a heterodimer with truncated pU_L34 (Figure S3B).

(G–I) The EM map of the NEC coat with four fitted hexameric SAXS-based models of soluble heterodimeric NEC is viewed from the side and sliced to remove density up to the "*6-2-6*" section passing through the map's center (G; for full hexamers, see Movie S10). The SAXS-derived model accounts for the EM density archways in all regions except the arch itself, thereby serving to validate the stoichiometry of our initial architectural model based on prediction of protein occupied volume. Tangential slices through the map at radii corresponding to MP (H) and MD (I) layers show that the "fitting search" using the SAXS-derived shape model is able to reproduce the characteristic features of the NEC coat (i.e., the two-layered arrangement) but also suggest that interactions between heterodimers occur predominantly across the 2-fold axis within both MD and MP layers, associated with pU_L31, and pU_L34, respectively.

the unit cell is composed of a hexamer of heterodimers ($[pU_L31/34]_6$). To independently validate the model, we characterized a soluble form of the PrV $pU_L31/34$ heterodimer (Figure S3) by small-angle X-ray scattering (SAXS) (Figure 6). We determined the shape of the soluble heterodimer by ab initio modeling from the 1D SAXS scattering curve (Figures 6A and 6B) (Franke and Svergun, 2009). The angular shape (87 nm^3 in volume, radius of gyration of 2.96 nm, and maximum dimension of 10.2 nm) was similar to that observed in the cryoEM sub-tomogram average as one side of the archway. To orient the soluble heterodimers within the cryoEM map, we carried out a fitting search using the SAXS model and sampled the full rotational range. By locally fitting multiple copies of the highest-scoring model into the cryoEM map, we were able to account for the cryoEM density as well as to reproduce its characteristic features (Figures 6C–6I and Movie S10). Together, this integrated modeling suggests that the NEC coat is composed of a ~10-nm-thick layer of interacting hexameric cores of NEC heterodimers in lateral self-association.

The densities of the MP layer leading to the membrane are not accounted for by the SAXS model (Figures 6D and 6E), consistent with it being the truncated sequence from pU_L34 that leads to the transmembrane domain (residues 180–240). The calculated mass from these residues (~40 kDa per hexamer) and the volume of the unaccounted density are congruent, confirming that the NEC coat consists only of pU_L31 and pU_L34, without direct contribution from any other viral or cellular factors, as was shown in vitro for HSV-1 (Bigalke et al., 2014). Previously, it has been reported that this membrane-connecting part of pU_L34 containing low-complexity/high-flexibility domains (Figure S3C) can be deleted and substituted by heterologous transmembrane-containing peptides (Paßvogel et al., 2014).

The NEC Structure Inherently Defines a Vesicle Size to Tightly Accommodate Viral Capsids

To unveil the architectural basis for its constrained curvature, we devised a simplified mathematical description of the NEC coat (Figure 7) and used this to produce a model of the coat that closely matches the measurements from the cryoFIB/ET average (Figure S4). The results confirm that the interplay of interactions within each layer and repeated heteromeric interactions between pU_L31 and pU_L34 define the curvature of the NEC coat.

Observation of a hexagonal NEC coat for the two alphaherpesviruses, PrV, as reported here, and HSV-1 (Bigalke et al.,

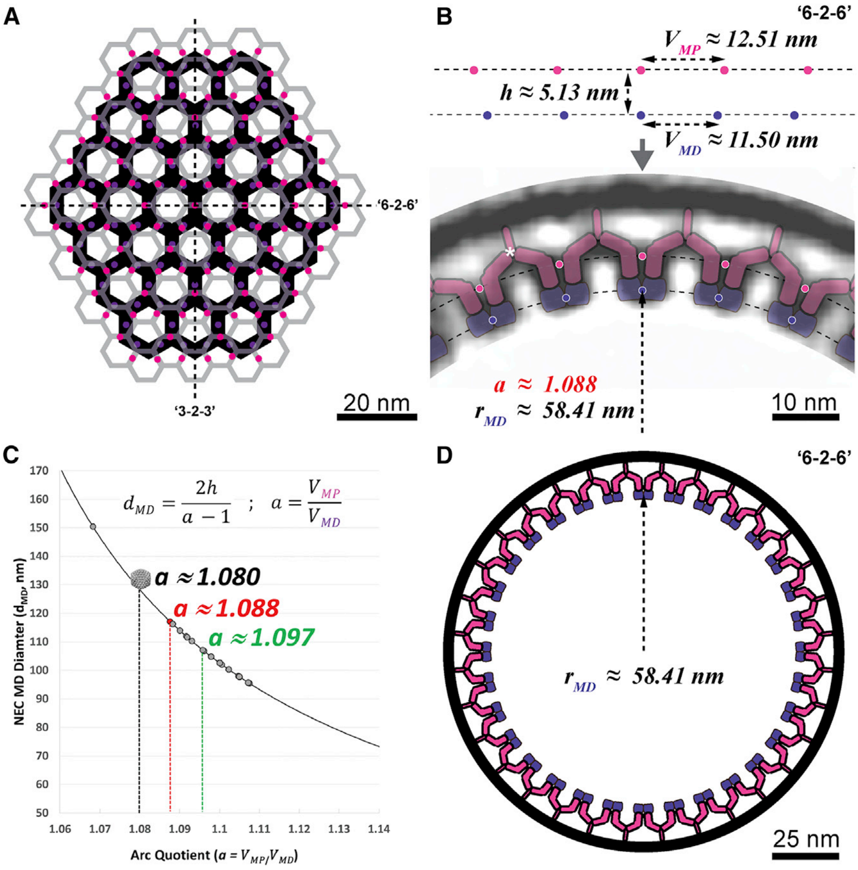

Figure 7. Architectural Basis for Constrained Curvature Formation

(A) A schematic model of the NEC where characteristic layers are represented as they appear in tangential slices (Figure 4B and Movie S8). Alignment of pink and purple points will result in formation of curvature essentially defined by the radial separation of each layer (parameter "*h*").

(B) A "*6-2-6*" cross-section view of the NEC coat average is modeled according to parameters (*h*, V_{MD}, and V_{MP}; compare Equation 1 in the Supplemental Experimental Procedures) measured from the experimental average. Magenta (MP) and purple (MD) circles highlight that hexagonal layers of characteristic repeat distances (V_{MD} and V_{MP}) interact via the pU$_L$31/34 heterodimer interface to induce a defined curvature. The basis for determination of the exact radial position of the two layers is given in Figure S4, and an arch keystone is marked with a white asterisk.

(C) The NEC coat diameter between opposite MD layers (d_{MD}) is plotted as a function of *a* (the arc quotient), and experimentally determined vesicle sizes from cryoFIB-prepared samples are shown as colored circles. The vesicle modeled in (B) is represented in red (see also red asterisk in Figures 3A–3C), while the mean vesicle size is indicated in green and a coated capsid by the respective symbol in gray, each with their respective *a* values. These values are plotted assuming that V_{MD} is constant, while V_{MP} is hypothesized to vary, owing to flexibility within coat.

(D) The resulting model in the context of an entire vesicle cross-section produced by extrapolation as described in the Supplemental Experimental Procedures. pU$_L$34 (magenta) and pU$_L$31 (purple) make up the MP and MD layers, respectively.

2014), suggests that interactions occurring at 2-fold and 3-fold axes of the MD (pU$_L$31) layer are likely evolutionarily conserved (lattice spacing ~11 nm in both cases). Interestingly, artificially membrane-tethered pU$_L$31 oligomers, i.e., in the absence of the native membrane tether pU$_L$34, did not show any regular/hexagonal pattern (Lorenz et al., 2015). Thus, by mutually confining the position of each of the six NEC heterodimers in space, the role of the pU$_L$34 membrane-connecting region is critically central in determining structural properties of the MP layer and thereby the curvature of the NEC coat. However, the distribution of vesicle sizes suggests that the NEC coat does not function as two rigidly imposed layers, i.e., it is not crystalline. While constrained in space by arch-forming interactions, the range of curvatures, vesicle sizes, and shapes observed (Figures 3D and 7C), starting from a planar NEC coat at initial budding sites (Figure 1B), is mediated by a high degree of flexibility in the membrane-connecting region of the coat.

In our experiments, we confirmed that, at artificially high local concentrations, in cells under constitutive expression (or in incubated vesicles), NECs alone can spontaneously form a coat and are able to mediate a complex process that involves induction of membrane curvature, vesicle budding, and scission. However, our observations at concentrations typical of the native situation, i.e., in infected cells, suggest that, in two-thirds of the perinuclear vesicles, the initial nucleation of pU$_L$31/34 heterodimers to form the NEC coat depended on presence of the capsid cargo (Figure 1).

In the cellular context, the NEC coat has the ability to form uniformly sized coated vesicles independent of the capsid cargo (ILVs in HSV-1-infected cells, Figures 1E and 3) (Klupp et al., 2007). A higher variability in curvature and hence size and shape has been observed in artificial model vesicles using partially truncated pU$_L$31/34 constructs without the genuine membrane anchor (Bigalke et al., 2014; Lorenz et al., 2015). The heteromeric combination of both pU$_L$31 and pU$_L$34 and their arrangement as hexamers as a result of the arch-forming interactions yield a structural environment (i.e., the inner surface of the coat) conceivably central to the task of selectively and efficiently recruiting and transporting the viral capsid. Thus, we propose that, while membrane-anchored pU$_L$31 is able to drive budding on its own (Lorenz et al., 2015), formation of vesicles of a curvature tailored specifically to the herpesviral capsid requires pU$_L$34.

Finally, we found that NEC coat assembly in situ produces vesicles of a size closely approximating but being somewhat smaller than capsids (Figures 3D and 7C). This size distribution in the absence of the capsid cargo suggests that it is most likely the capsid itself that determines the minimum diameter of an enveloped capsid (Figure 7C, gray capsid symbol), as the NEC coat appears to inherently favor a slightly higher curvature and thus

smaller vesicle size. Concomitantly, this ensures a very tight fit and interaction between the NEC coat and the capsid, leading to a cargo vesicle of the smallest possible size given the components involved.

Our current functional model of capsid envelopment at the INM can be summarized as follows: sparsely distributed NEC heterodimers form a planar layer at the INM, either spontaneously or initiated by cargo/capsid docking. At this point, the lattice already shows the ~11 nm spacing of the hexagonal MD/pU_L31 layer (Bigalke et al., 2014). Driven potentially by structural changes in the pU_L34 region during the concomitant formation of a second hexagonal layer (MP), budding of the INM into the perinuclear space is induced. New NEC heterodimers are recruited at the rim of the coat until it reaches its curvature limit through interaction of the MD/MP layers at a size to precisely envelope a herpesviral capsid.

Our insights into the molecular mechanism of remodeling the nuclear envelope for viral nuclear egress provide a molecular template by which nucleo-cytoplasmic transport can occur. The precise architecture of the NEC defines vesicles with a specific size, allowing an efficient but highly controlled method of egress. This mechanism allows the transport of cargoes with minimal disruption to the INM, a feature essential for the egress of equivalent cellular cargoes. Crucially, membrane-anchored proteins mediating a divergent process would pre-assemble with a potentially modular cargo-recruitment adaptor to form heterodimeric units capable of forming lateral, self-assembling lattices. Formation of curvature by this lattice is then a pre-requisite for envelopment of egressing cargoes, features that would be evident when investigated in vitro. High-resolution structures of NEC from different herpesvirus species have now emerged that may reveal a common structural homology to cargo recruitment at the INM (Bigalke and Heldwein, 2015; Leigh et al., 2015; Lye et al., 2015; Walzer et al., 2015). Interestingly, pU_L31 contains a conserved zinc-finger motif essential for vesicle formation and NEC function (Zeev-Ben-Mordehai et al., 2015). Using the curved lattice structure described here as a model for fitting the atomic structure of the NEC heterodimer (Zeev-Ben-Mordehai et al., 2015), we have defined exact interaction surfaces that could be used as a further constraint for structural and functional homology modeling of putative cellular counterparts.

Recently, it has been shown that TorsinA AAA+ ATPase is activated in a complex with type II membrane protein LAP1 at the INM (Brown et al., 2014; Sosa et al., 2014). As speculated in McCullough and Sundquist (2014), that complex might also be involved in perinuclear vesicle formation during transport of ribonucleoprotein particles in *Drosophila* cells in which TorsinA has been shown to promote INM scission (Jokhi et al., 2013). This complex and the NEC might share molecular attributes like the zinc-finger motif coming from a common ancestor when (and if) herpesvirus has hijacked this pathway in evolution (Forterre and Prangishvili, 2013). However, there are many issues in determining common ancestors for protein structures, including the increased mutation rate of viral genomes (Abroi and Gough, 2011). A next practical step to analyze that further might be to apply cryoEM also in vesicle-accumulating TorsinA-mutated cells described in Jokhi et al. (2013). This imaging technique is the sole method that can elucidate the direct presence of a (protein) coat along a membrane unequivocally as it avoids artifacts by chemical fixation and heavy metal staining and can be combined with immunostaining (Karreman et al., 2011). Finding a coat of TorsinA-LAP1 complexes, or any other players implicated in nuclear egress, might then suggest a similar mechanism of vesicle formation in a general nucleo-cytoplasmic transport pathway of large cargo, as described here for nuclear egress of herpesviral capsids.

Concluding Remarks

The described NEC coat architecture is an elegant solution for induction of membrane curvature based solely on the formation of a highly defined lattice of heterodimer interactions. This is reminiscent of virus budding at the plasma membrane, e.g., HIV (Sundquist and Kräusslich, 2012). However, the NEC targets the INM, a membrane for which no other vesicle transport has yet been mechanistically fully elucidated (Jokhi et al., 2013). Furthermore, while most cellular vesicle formation processes involve a dedicated cellular scission machinery and consume energy in form of ATP or similar, the NEC (1) appears capable of autoscission by continuing assembly of NEC units on the inside of the forming vesicle (Bigalke et al., 2014) and (2) requires at least under in vitro conditions no external energy input for both membrane budding and scission (Lorenz et al., 2015). Elucidating the unique features of the binary $pU_L31/34$ vesicle formation machinery might provide the blueprint for designing vesicles of highly defined sizes or specific volumes to be used in pharmaceutical and nanobiotechnological applications. Moreover, the characterization of the nature of the viral cargo packing system at the INM opens the search for the respective cellular counterparts and molecular determinants mediating nuclear egress of cellular large cargo, including ribonucleoprotein particles (Hatch and Hetzer, 2014; Jokhi et al., 2013).

EXPERIMENTAL PROCEDURES

Cryo-Electron Microscopy of Vitreous Sections

Sixteen hours after infection with herpes simplex virus 1 (HSV-1) strain K26GFP (Desai and Person, 1998) at a multiplicity of infection of 10, trypsinized African green monkey kidney cells (Vero cells, strain CCL-81; ATCC) or proteinase K-treated porcine epithelial-like embryonic EFN-R kidney cells stably co-expressing PrV pU_L31 and pU_L34, the latter fused with GFP (cell line designated as BK/EFN/$U_L31/34$ gfp, here abbreviated to BK, catalog No. RIE 1083 of the Collection of Cell Lines in Veterinary Medicine at the FLI, Greifswald-Insel Riems, Germany) (Hagen et al., 2012; Klupp et al., 2007), were physically fixed and analyzed. Cryo-immobilization was performed by high-pressure freezing followed by cryo-electron microscopy of vitreous sections (CEMOVIS), essentially as described in Hagen and Grünewald (2008). Further details are available in the Supplemental Experimental Procedures.

Live-Cell Three-Dimensional Structured Illumination Microscopy

BK cells were grown on high-precision 22 × 22 mm No. 1.5H glass coverslips (Marienfeld Superior) or in μ-Dish, high glass bottom 35-mm dishes (Ibidi GmbH, Martinsried, Germany) to a confluency of ~70%–80% in 10% (w/v) fetal bovine serum in Dulbecco's modified Eagle medium (DMEM; GIBCO-Invitrogen). Before imaging, the medium was replaced with pre-warmed Opti-MEM (GIBCO-Invitrogen). Three-dimensional structured illumination microscopy (3D-SIM) (Gustafsson et al., 2008) on live-cell samples was performed using an OMX V3 Blaze system (Applied Precision, GE Healthcare) (Strauss et al., 2012) equipped with a 60×/1.42 NA PlanApo oil-immersion objective (Olympus), a 488-nm diode laser with standard filter sets, and

Edge sCMOS cameras (PCO). Further details are available in the Supplemental Experimental Procedures.

CryoEM/T of Lamellae Produced by CryoFIB in a Dual-Beam Scanning Electron FIB-SEM Cryo-Microscope

Standard 3.05 mm electron microscopy 200 mesh gold grids covered with a perforated carbon foil (R2/1; Quantifoil Micro Tools GmbH, Jena, Germany) were hydrophilised in a PDC-002 plasma cleaner (Harrick Plasma, Ithaca, NY, USA). BK cells were grown on these grids in DMEM supplemented with 10% (w/v) fetal calf serum and 1% (v/v) PSN Antibiotic Mixture (GIBCO-Invitrogen), essentially as performed for 3D-SIM and soft X-ray microscopy samples. After 2 days of incubation (37°C, 5% CO_2) in plastic microscope slide growth chambers (μ-slide 2 × 9 well; Ibidi GmbH) and light microscopic screening for optimal growth, cells were cryo-immobilized by plunge freezing, as described in Hagen et al., 2012).

CryoFIB was essentially performed as recently described (Engel et al., 2015). It is detailed in the Supplemental Experimental Procedures.

For tomography of the cryoFIB lamellae, a Tecnai G2 Polara transmission electron microscope (FEI) equipped with a field emission gun operated at 300 kV, a GIF 2002 post-column energy filter (Gatan, Pleasanton, CA), and a 2048 × 2048 Gatan Multiscan CCD camera were used. Tomographic tilt-series acquisition under low-dose conditions (10 tilt series out of 14 lamellae, tilt range: −55° to 59°, cumulative dose: 110 electrons per Å^2) was controlled by SerialEM (Mastronarde, 2005). Tilt-series images were recorded at 3° tilt increments, with −6 μm defocus, at an object pixel size of 0.57 nm.

For alignment of the tilt-series projections, small spherical cellular features or ice contaminants were employed as tracking markers, or patch tracking following the routine in the Etomo GUI of IMOD was applied. Tomograms were reconstructed using weighted back projections, and visualization was performed with Amira 5.2 (FEI).

Sub-Tomogram Averaging and Modeling of CryoFIB/ET Data

Sub-tomogram averaging was carried out using the PEET package applying constrained cross-correlation (CCC) (Briggs, 2013; Nicastro et al., 2006). Details of data processing, integrated analysis, and model building are available in the Supplemental Experimental Procedures.

Soluble NEC Preparation

Details on the construction of a soluble NEC expression vector are provided in the Supplemental Experimental Procedures. *Escherichia coli* BL21 (DE3) transformed with pETDuet::U_L34(1–179)::U_L31-NLS was grown to saturation overnight at 25°C in LB medium containing ampicillin (100 μg ml^{-1}). An aliquot of overnight culture was diluted 1/20 in medium containing ampicillin and was grown at 37°C to an OD_{600} of 0.6, at which time expression was induced by addition of isopropyl-D- galactoside (IPTG) to a final concentration of 1 mM. Cells were incubated for a further 4.5 hr at 25°C before being harvested by centrifugation (3,500 × *g*, 10 min, 4°C), and stored at −20°C.

Cells were re-suspended in buffer A (PBS pH 7.4); supplemented with 2,500 units DNase 1, 0.2 mg ml^{-1} lysozyme, 5 mM $MgSO_4$, and 1% protease inhibitor cocktail (Sigma); and lysed by sonication. The supernatant was clarified by centrifugation (45,000 × *g*, 30 min, 4°C).

The complex was purified by metal affinity chromatography (co-sepharose 6-fast flow) followed by size-exclusion chromatography in buffer B (10 mM Tris-HCl [pH 7.4], 75 mM NaCl, 3 mM DTT). Purified proteins were analyzed by SDS-PAGE.

Small-Angle X-Ray Scattering Data Collection, Processing, and Analysis

SAXS data for soluble NEC were collected on the BM29 beamline at the ESRF synchrotron (Grenoble, France). Details are available in the Supplemental Experimental Procedures.

ACCESSION NUMBERS

The sub-tomographic reconstruction has been deposited in the Electron Microscopy Data Bank (EMDB) at PDBe (http://www.ebi.ac.uk/pdbe/emdb/) as EMD-3215.

SUPPLEMENTAL INFORMATION

Supplemental Information includes Supplemental Experimental Procedures, four figures, and ten movies and can be found with this article online at http://dx.doi.org/10.1016/j.cell.2015.11.029.

AUTHOR CONTRIBUTIONS

All authors intellectually conceived the experiments. C.H. performed CEMOVIS, cryoXM/T, and cryoFIB; K.C.D. did cryoET sub-tomogram analysis and integrated modeling; T.Z.-B.-M. provided SAXS data and models; and M.G. and J.B.B. imaged cells by live 3D-SIM. Furthermore, C.W., B.G.K., C.A.S., F.J.B.B., J.C., S.W., P.G., S.R., K.H., J.D., and B.A. performed experiments; D.V. processed and analyzed data; C.H., K.C.D., T.Z.-B.-M., M.G., J.B.B., T.C.M., and K.G. wrote the manuscript, and all authors commented on it.

ACKNOWLEDGMENTS

We thank Wolfram Antonin (Tübingen) for critical discussion and Marion Jasnin, Tim Laugks, Miroslava Schaffer, and Margarete Schüler for help with cryoFIB in Martinsried. This work was supported by a Wellcome Trust Senior Research Fellowship to K.G. (090895/Z/09/Z), German Research Council grants DFG GR1990/2 to K.G. and DFG Me 854/12-1 to T.C.M., a Leverhulme Trust grant to K.G. (RPG-2012-519), a Wellcome Trust JIF award (060208/Z/00/Z) and a Wellcome Trust equipment grant (093305/Z/10/Z) to the Oxford Particle Imaging Centre, the Wellcome Trust core award 090532/Z/09/Z to the Wellcome Trust Centre for Human Genetics, the Wellcome Trust Strategic Award 091911 supporting advanced microscopy at Micron Oxford, a Wellcome Trust PhD studentship to M.G., a São Paulo Research Foundation Fellowship to J.C. (Fapesp 2013/24972-1), and a DFG Fellowship BO 4158/1-1 to J.B.B. We acknowledge the Helmholtz-Zentrum Berlin electron storage ring BESSY II for provision of synchrotron radiation at beamline U41-FSGM. The research leading to these results has received funding from the European Community's Seventh Framework Programme (FP7/2007-2013) under BioStruct-X (Grant agreement nos. 283570 and 226716).

Received: June 1, 2015
Revised: September 11, 2015
Accepted: November 6, 2015
Published: December 17, 2015

REFERENCES

Abroi, A., and Gough, J. (2011). Are viruses a source of new protein folds for organisms? - Virosphere structure space and evolution. BioEssays *33*, 626–635.

Bigalke, J.M., and Heldwein, E.E. (2015). Structural basis of membrane budding by the nuclear egress complex of herpesviruses. EMBO J. *34*, 2921–2936.

Bigalke, J.M., Heuser, T., Nicastro, D., and Heldwein, E.E. (2014). Membrane deformation and scission by the HSV-1 nuclear egress complex. Nat. Commun. *5*, 4131.

Briggs, J.A.G. (2013). Structural biology *in situ*–the potential of subtomogram averaging. Curr. Opin. Struct. Biol. *23*, 261–267.

Brown, R.S.H., Zhao, C., Chase, A.R., Wang, J., and Schlieker, C. (2014). The mechanism of Torsin ATPase activation. Proc. Natl. Acad. Sci. USA *111*, E4822–E4831.

Desai, P., and Person, S. (1998). Incorporation of the green fluorescent protein into the herpes simplex virus type 1 capsid. J. Virol. *72*, 7563–7568.

Dubochet, J. (2012). Cryo-EM–the first thirty years. J. Microsc. *245*, 221–224.

Engel, B.D., Schaffer, M., Cuellar, L.K., Villa, E., Plitzko, J.M., and Baumeister, W. (2015). Native architecture of the *Chlamydomonas* chloroplast revealed by *in situ* cryo-electron tomography. eLife *4*, e04889.

Forterre, P., and Prangishvili, D. (2013). The major role of viruses in cellular evolution: facts and hypotheses. Curr. Opin. Virol. *3*, 558–565.

Franke, D., and Svergun, D.I. (2009). DAMMIF, a program for rapid *ab-initio* shape determination in small-angle scattering. J. Appl. Cryst. *42*, 342–346.

Funk, C., Ott, M., Raschbichler, V., Nagel, C.H., Binz, A., Sodeik, B., Bauerfeind, R., and Bailer, S.M. (2015). The herpes simplex virus protein pU_L31 escorts nucleocapsids to sites of nuclear egress, a process coordinated by its N-terminal domain. PLoS Pathog. *11*, e1004957.

Gustafsson, M.G.L., Shao, L., Carlton, P.M., Wang, C.J.R., Golubovskaya, I.N., Cande, W.Z., Agard, D.A., and Sedat, J.W. (2008). Three-dimensional resolution doubling in wide-field fluorescence microscopy by structured illumination. Biophys. J. *94*, 4957–4970.

Hagen, C., and Grünewald, K. (2008). Microcarriers for high-pressure freezing and cryosectioning of adherent cells. J. Microsc. *230*, 288–296.

Hagen, C., Guttmann, P., Klupp, B., Werner, S., Rehbein, S., Mettenleiter, T.C., Schneider, G., and Grünewald, K. (2012). Correlative VIS-fluorescence and soft X-ray cryo-microscopy/tomography of adherent cells. J. Struct. Biol. *177*, 193–201.

Hatch, E., and Hetzer, M. (2014). Breaching the nuclear envelope in development and disease. J. Cell Biol. *205*, 133–141.

Hoenger, A. (2014). High-resolution cryo-electron microscopy on macromolecular complexes and cell organelles. Protoplasma *251*, 417–427.

Jokhi, V., Ashley, J., Nunnari, J., Noma, A., Ito, N., Wakabayashi-Ito, N., Moore, M.J., and Budnik, V. (2013). Torsin mediates primary envelopment of large ribonucleoprotein granules at the nuclear envelope. Cell Rep. *3*, 988–995.

Karreman, M.A., van Donselaar, E.G., Gerritsen, H.C., Verrips, C.T., and Verkleij, A.J. (2011). VIS2FIX: a high-speed fixation method for immuno-electron microscopy. Traffic *12*, 806–814.

Klupp, B.G., Granzow, H., Fuchs, W., Keil, G.M., Finke, S., and Mettenleiter, T.C. (2007). Vesicle formation from the nuclear membrane is induced by coexpression of two conserved herpesvirus proteins. Proc. Natl. Acad. Sci. USA *104*, 7241–7246.

Leigh, K.E., Sharma, M., Mansueto, M.S., Boeszoermenyi, A., Filman, D.J., Hogle, J.M., Wagner, G., Coen, D.M., and Arthanari, H. (2015). Structure of a herpesvirus nuclear egress complex subunit reveals an interaction groove that is essential for viral replication. Proc. Natl. Acad. Sci. USA *112*, 9010–9015.

Lorenz, M., Vollmer, B., Unsay, J.D., Klupp, B.G., García-Sáez, A.J., Mettenleiter, T.C., and Antonin, W. (2015). A single herpesvirus protein can mediate vesicle formation in the nuclear envelope. J. Biol. Chem. *290*, 6962–6974.

Loret, S., Guay, G., and Lippé, R. (2008). Comprehensive characterization of extracellular herpes simplex virus type 1 virions. J. Virol. *82*, 8605–8618.

Lučić, V., Förster, F., and Baumeister, W. (2005). Structural studies by electron tomography: from cells to molecules. Annu. Rev. Biochem. *74*, 833–865.

Lye, M.F., Sharma, M., El Omari, K., Filman, D.J., Schuermann, J.P., Hogle, J.M., and Coen, D.M. (2015). Unexpected features and mechanism of heterodimer formation of a herpesvirus nuclear egress complex. EMBO J. *34*, 2937–2952.

Marko, M., Hsieh, C., Schalek, R., Frank, J., and Mannella, C. (2007). Focused-ion-beam thinning of frozen-hydrated biological specimens for cryo-electron microscopy. Nat. Methods *4*, 215–217.

Mastronarde, D.N. (2005). Automated electron microscope tomography using robust prediction of specimen movements. J. Struct. Biol. *152*, 36–51.

McCullough, J., and Sundquist, W.I. (2014). Putting a finger in the ring. Nat. Struct. Mol. Biol. *21*, 1025–1027.

McMahon, H.T., and Gallop, J.L. (2005). Membrane curvature and mechanisms of dynamic cell membrane remodelling. Nature *438*, 590–596.

Mettenleiter, T.C., Müller, F., Granzow, H., and Klupp, B.G. (2013). The way out: what we know and do not know about herpesvirus nuclear egress. Cell. Microbiol. *15*, 170–178.

Mou, F., Wills, E., and Baines, J.D. (2009). Phosphorylation of the U_L31 protein of herpes simplex virus 1 by the U_S3-encoded kinase regulates localization of the nuclear envelopment complex and egress of nucleocapsids. J. Virol. *83*, 5181–5191.

Nicastro, D., Schwartz, C., Pierson, J., Gaudette, R., Porter, M.E., and McIntosh, J.R. (2006). The molecular architecture of axonemes revealed by cryoelectron tomography. Science *313*, 944–948.

Panté, N., and Kann, M. (2002). Nuclear pore complex is able to transport macromolecules with diameters of about 39 nm. Mol. Biol. Cell *13*, 425–434.

Paßvogel, L., Janke, U., Klupp, B.G., Granzow, H., and Mettenleiter, T.C. (2014). Identification of conserved amino acids in pU_L34 which are critical for function of the pseudorabies virus nuclear egress complex. J. Virol. *88*, 6224–6231.

Rigort, A., Bäuerlein, F.J.B., Villa, E., Eibauer, M., Laugks, T., Baumeister, W., and Plitzko, J.M. (2012). Focused ion beam micromachining of eukaryotic cells for cryoelectron tomography. Proc. Natl. Acad. Sci. USA *109*, 4449–4454.

Rose, A., and Schlieker, C. (2012). Alternative nuclear transport for cellular protein quality control. Trends Cell Biol. *22*, 509–514.

Schekman, R., and Orci, L. (1996). Coat proteins and vesicle budding. Science *271*, 1526–1533.

Schermelleh, L., Heintzmann, R., and Leonhardt, H. (2010). A guide to super-resolution fluorescence microscopy. J. Cell Biol. *190*, 165–175.

Sharma, M., Bender, B.J., Kamil, J.P., Lye, M.F., Pesola, J.M., Reim, N.I., Hogle, J.M., and Coen, D.M. (2015). Human cytomegalovirus U_L97 phosphorylates the viral nuclear egress complex. J. Virol. *89*, 523–534.

Sosa, B.A., Demircioglu, F.E., Chen, J.Z., Ingram, J., Ploegh, H.L., and Schwartz, T.U. (2014). How lamina-associated polypeptide 1 (LAP1) activates Torsin. eLife *3*, e03239.

Speese, S.D., Ashley, J., Jokhi, V., Nunnari, J., Barria, R., Li, Y., Ataman, B., Koon, A., Chang, Y.T., Li, Q., et al. (2012). Nuclear envelope budding enables large ribonucleoprotein particle export during synaptic Wnt signaling. Cell *149*, 832–846.

Strauss, M.P., Liew, A.T.F., Turnbull, L., Whitchurch, C.B., Monahan, L.G., and Harry, E.J. (2012). 3D-SIM super resolution microscopy reveals a bead-like arrangement for FtsZ and the division machinery: implications for triggering cytokinesis. PLoS Biol. *10*, e1001389.

Sundquist, W.I., and Kräusslich, H.G. (2012). HIV-1 assembly, budding, and maturation. Cold Spring Harb. Perspect. Med. *2*, a006924.

Walzer, S.A., Egerer-Sieber, C., Sticht, H., Sevvana, M., Hohl, K., Milbradt, J., Muller, Y.A., and Marschall, M. (2015). Crystal structure of the human cytomegalovirus pUL50-pUL53 core nuclear egress complex provides insight into a unique assembly scaffold for virus-host protein interactions. J. Biol. Chem. *290*, 27452–27458.

Webster, B.M., Colombi, P., Jäger, J., and Lusk, C.P. (2014). Surveillance of nuclear pore complex assembly by ESCRT-III/Vps4. Cell *159*, 388–401.

Wild, P., Leisinger, S., de Oliveira, A.P., Schraner, E.M., Kaech, A., Ackermann, M., and Tobler, K. (2015). Herpes simplex virus 1 U_s3 deletion mutant is infective despite impaired capsid translocation to the cytoplasm. Viruses *7*, 52–71.

Zeev-Ben-Mordehai, T., Hagen, C., and Grünewald, K. (2014). A cool hybrid approach to the herpesvirus ‘life’ cycle. Curr. Opin. Virol. *5*, 42–49.

Zeev-Ben-Mordehai, T., Weberruß, M., Lorenz, M., Cheleski, J., Hellberg, T., Whittle, C., El Omari, K., Vasishtan, D., Dent, K.C., Harlos, K., et al. (2015). Crystal structure of the herpesvirus nuclear egress complex provides insights into inner nuclear membrane remodeling. Cell Rep. *13*. Published online December 17, 2015. http://dx.doi.org/10.1016/j.celrep.2015.11.008.

Supplemental Figures

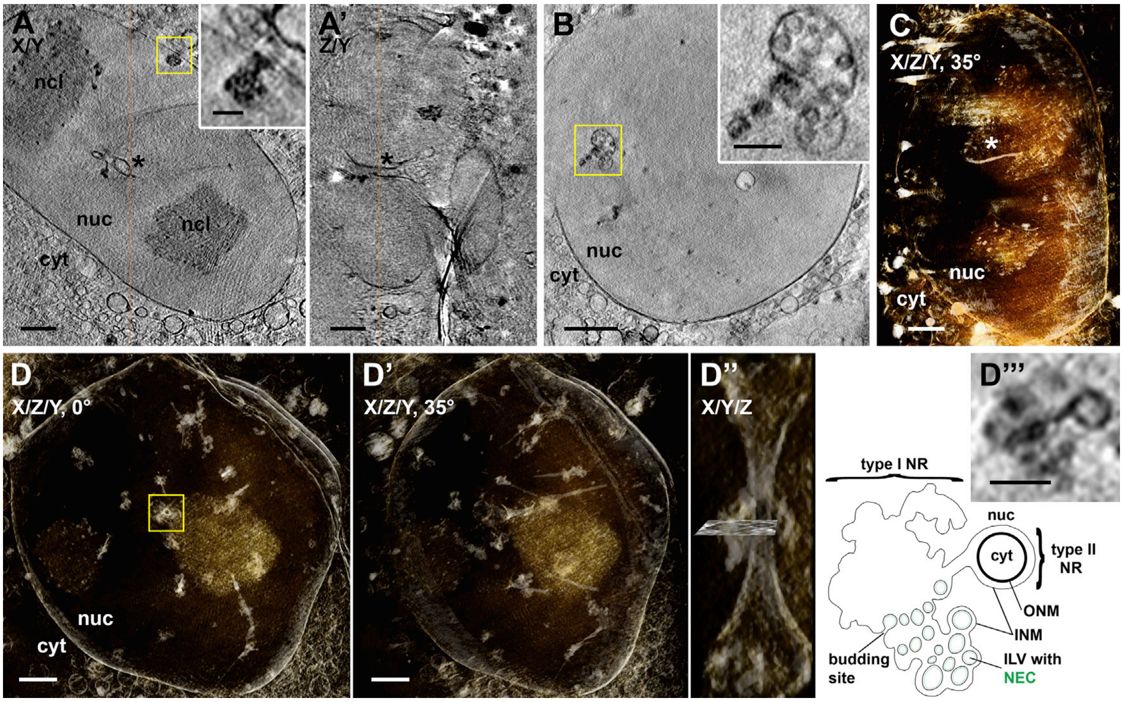

Figure S1. Nuclear Structure in PrV pU$_L$31/pU$_L$34-GFP Co-expressing Cells, Related to Figure 2

(A–D) Soft X-ray cryo-microscopy/tomography (cryoXM/T) of BK cells (A) Slice of a soft X-ray tomogram, taken with a 40 nm zone plate objective (yellow box and a magnification of a similar slice in the same area: inset). CryoXM/T directly images frozen-hydrated unstained samples and gives about five times higher resolution than 3D-SIM while still allowing the entire nucleus to be observed. Note the high absorption contrast, revealing details of nuclear substructure like nucleoli. Infoldings of the INM were clearly detectable as racemose, tree- or mushroom-shaped clusters, mostly near the nuclear envelope (yellow box and inset). These clusters correlated with fluorescent 'speckles' containing pU$_L$34-GFP/NEC (Hagen et al., 2012). (A') Side view of the same tomogram. Asterisks in (A) and (A') mark a tubular invagination of both nuclear envelope membranes running through the nucleus (orange lines: sectional planes). We observed such infoldings in almost all nuclei (28 out of 31; from 28 tomograms). The tubes were often the origin of INM-infoldings. Such infoldings of the INM or of both membranes of the nuclear envelope have been observed in many eukaryotic cells, and are termed nucleoplasmic reticulum (NR) type I or type II, respectively (Malhas et al., 2011). (B) Cells grown only 15 hr on grid (instead of 2 days) had less tubes but exhibited more vesicle-like infoldings of the INM. In larger vesicles, smaller spherical vesicles were detected, exhibiting the size of ILVs (yellow box and an un-binned magnification of a similar slice in the same area: inset). (C) Tubular invaginations of the nuclear membranes, here visualized with inverse contrast in a rendered volume of a tomographic reconstruction, occurred only rarely in non-transfected control cells (asterisk), suggesting induction/enhancement of their formation by co-expression of the NEC components pU$_L$31 and pU$_L$34. (D) Tubular infoldings of the entire nuclear envelope crossed the nucleus preferentially, but not invariably, along its smallest extension (D', Movie S4). The side view of these tubes revealed the interconnection of type I and type II NR (D''), as shown in detail for one sub-volume of the tomographic reconstruction (yellow box in D, slice in D'', zoom and schematic interpretation in D'''). Scale bar is 2 μm (A-D') and 500 nm (insets; D'''). cyt, cytoplasm; ILV, intraluminal vesicle; INM, inner nuclear membrane; ncl, nucleolus; NEC, nuclear egress complex; NR, nucleoplasmic reticulum; nuc, nucleus; ONM, outer nuclear membrane

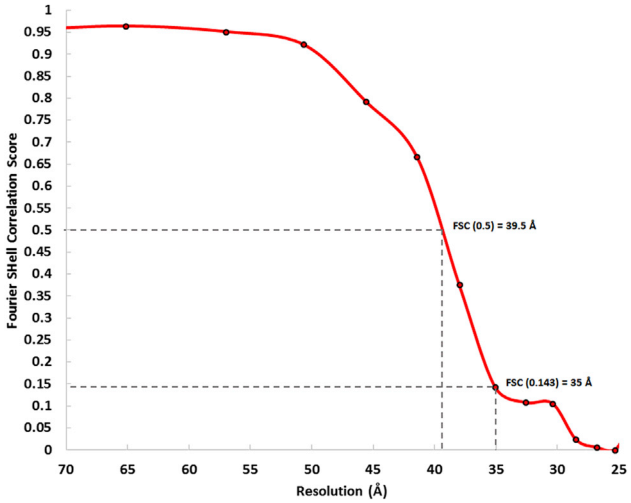

Figure S2. Resolution Assessment of the NEC Coat Map, Related to Figure 4

Fourier shell correlation calculated between two half-datasets of 150 particles each. The resolution of the map is estimated to be between 35 and 40 Å. At a nominal defocus of -6 μm the first node of the CTF is expected at 1/34 Å^{-1}.

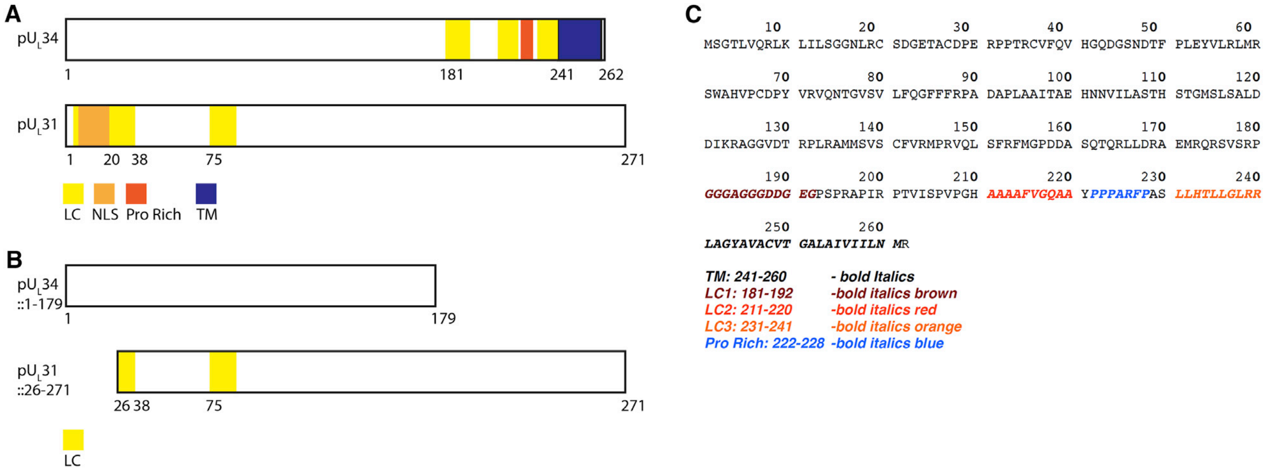

Figure S3. Overview of Protein Constructs, Related to Figure 6

(A) Sequence motifs for full-length pU$_L$31 (Uniprot: G3G955) and pU$_L$34 (Uniprot: G3G8X8) (PrV) are illustrated. pU$_L$31 exhibits a N-terminal nuclear localization signal (NLS, orange), while pU$_L$34 contains a C-terminal trans-membrane (TM, blue) domain (LC: low complexity domain, yellow; Pro Rich: proline rich domain, red). The NEC coat produced by co-expression and in situ assembly of the pU$_L$31/34 heterodimer (including a C-terminal GFP-tag at pU$_L$34) in porcine kidney cells was visualized by 3D-SIM, cryoXM/T, CEMOVIS and cryoFIB/ET. The GFP tag did not (substantially) affect membrane interactions/curvature as concluded from the following two lines of evidence: (i) Nuclei of HSV-1 infected host cells (native full length pU$_L$31/pU$_L$34 without any fluorescent tag) exhibited similar intraluminal vesicles (and NEC coats) as the cell model with pU$_L$34-GFP. (ii) The GFP-cryoEM densities, that topologically face the perinuclear space, were not structured regularly and did not follow the hexameric structure of the membrane-tethering/transmembrane C-terminal part of pU$_L$34 (Figure 4A, Movie S7).
(B) Truncated recombinant constructs (pU$_L$31ΔNLS and pU$_L$34t179) are illustrated as used for bacterially co-expression for SAXS experiments, as well as for the subsequent SAXS-EM fitting search. The soluble NEC expressed for SAXS measurements did not contain the C terminus of pUL34, and hence no GFP.
(C) The amino acid sequence of pU$_L$34 (PrV, Uniprot: G3G8X8) is shown and highlights five regions of interest. The transmembrane domain (TM) is colored black, and three low complexity regions (presumably highly flexible), are colored brown, red, and orange. These are in addition to a proline rich region (colored blue).

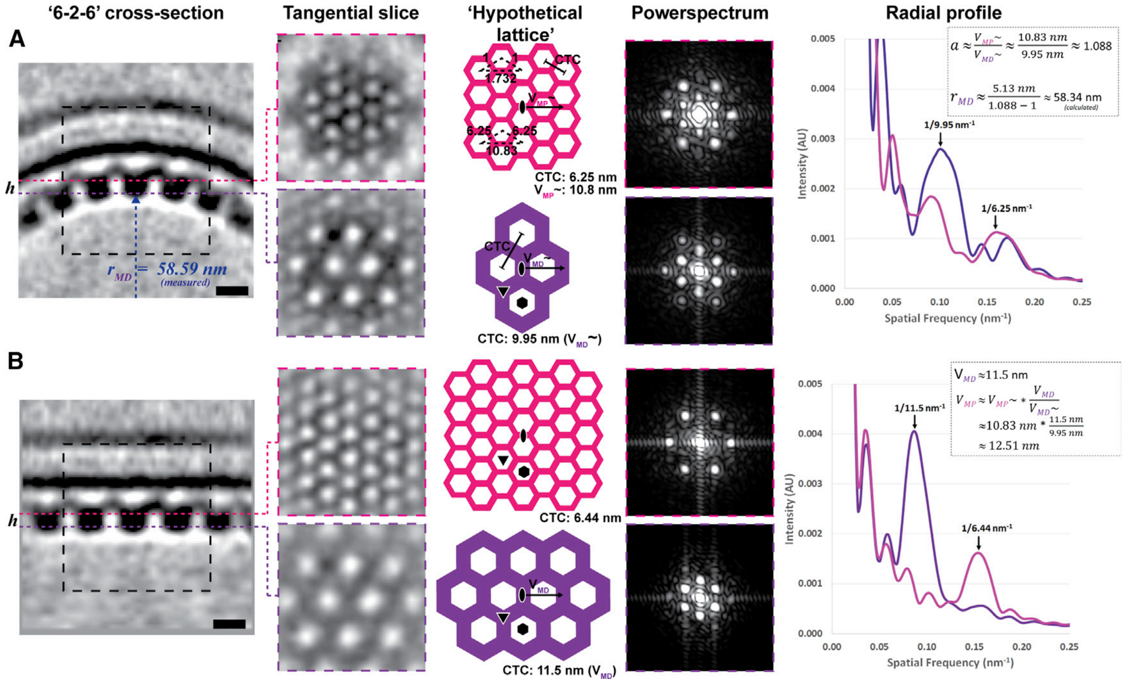

Figure S4. Characteristic Layers of the NEC CryoEM Map and Curvature Modeling, Related to Figure 7

(A and B) The ‘raw’ NEC coat average map (A; Movie S7), and radially projected NEC coat map (B) are centered on a 2-fold axis, and viewed along a *‘6-2-6’* cross-section. Any given 2-fold axis is neighbored by only two 6-fold axes, and will therefore participate in only one *‘6-2-6’* plane – the direction of curvature in the present model. For each map (A and B), tangential slices at vesicle membrane distal (MD, lower, purple) and vesicle membrane proximal (MP, upper, magenta) layers are shown. These layers are separated radially by 5.13 nm (parameter ‘*h*’) (Figure 7). Layers have characteristic hexagonal (‘honeycomb’) patterns of protein density (Movie S9). The MD layer density at 3.5 nm resolution does not reveal clearly distinct aggregates of globular domains, but instead appears as a continuous layer of protein density. The MP layer appears to be a distinct (more finely spaced) hexagonal lattice with each presumed pU_L34 unit appearing to form a vertex of the unit cell, with apparent connections to neighbors across the 2-fold axes. These layers are interpreted as ‘hypothetical lattices’ (colored appropriately) with characteristic center-to-center (CTC) distances (or ‘spacing’) as illustrated (not to scale). Observing the slices in reciprocal space as power spectra (i.e., squared amplitudes of structure factors) allowed detection of two primary reflections corresponding to ~10 nm (MD layer) and ~6 nm (MP layer, indicated by arrows), giving confirmation of the uniquely differing arrangement of densities between these layers and confirming that the MP lattice is offset by 30° in relation to the MD lattice. Radial average ‘profile’ curves for MD (purple) and MP (magenta) shown rightmost allow lattice parameters to be measured accurately. The relations annotated allow arc length parameters V_{MD} and V_{MP} to be approximated, and applied to estimate the vesicle radius (Figure 7C). Measurements from reflections observed from the curved NEC coat map (A) are inherently underestimated – owing to the curvature itself causing signal from multiple radial layers to contribute to the measurement, however the ratio of these is preserved and therefore used to estimate the arc quotient (‘*a*’) as shown. The MD lattice is established by pU_L31 interactions (at 2-fold and 3-fold interfaces) and are used as a ruler (providing the true V_{MD}), and therefore used to scale $V_{MP}^{\sim}$ as estimated from the curved NEC coat map. Calculations are shown as inset. Slices are 0.57 nm thick.

Cell

Supplemental Information

Structural Basis of Vesicle Formation at the Inner Nuclear Membrane

Christoph Hagen, Kyle C. Dent, Tzviya Zeev-Ben-Mordehai, Michael Grange, Jens B. Bosse, Cathy Whittle, Barbara G. Klupp, C. Alistair Siebert, Daven Vasishtan, Felix J. B. Bäuerlein, Juliana Cheleski, Stephan Werner, Peter Guttmann, Stefan Rehbein, Katja Henzler, Justin Demmerle, Barbara Adler, Ulrich Koszinowski, Lothar Schermelleh, Gerd Schneider, Lynn W. Enquist, Jürgen M. Plitzko, Thomas C. Mettenleiter, and Kay Grünewald

SUPPLEMENTAL EXPERIMENTAL PROCEDURES

Electron Cryo-Microscopy of Vitreous Sections - CEMOVIS

CEMOVIS is the 'gold standard' among sectioning methods for electron microscopy (Bleck et al., 2010; Gunkel et al., 2015; Mielanczyk et al., 2014). However, structural artefacts such as inhomogeneous compression and membrane crevasses (Figure 2B, asterisks) (Al-Amoudi et al., 2005; Bouchet-Marquis and Hoenger, 2011; Pierson et al., 2011), impaired higher-resolution analysis.

Compression due to the sectioning process was estimated based on the change in aspect ratio of HSV-1 capsids. A non-linear compression dependency on the nominal section feed similar to that described in (Han et al., 2008) was observed. Projection images from vitreous sections were corrected for this thickness-dependent compression along the cutting direction (knife marks) by rescaling and interpolation, and were low-pass filtered for an improved signal to noise ratio. The projection images of tomographic tilt series were aligned using a MATLAB-implementation (provided by Matthias Eibauer, University of Zurich, Department of Biochemistry, Switzerland) of marker-free image registration algorithms described in (Sorzano et al., 2009), in combination with the global alignment correction capabilities of IMOD (Kremer et al., 1996; Mastronarde, 2008) to account for electron beam-induced movements of the vitreous section (Hsieh et al., 2006). Tomographic reconstructions were corrected for compression according to the model given in (Al-Amoudi et al., 2005), including lateral re-positioning of all X/Y-slices in the Z-direction, using MATLAB scripts provided by Lars-Anders Carlson (University of California, Department of Molecular & Cell Biology, Berkeley, CA). If not stated otherwise, CEMOVIS tomograms presented here are binned with a kernel of 2x2x2 and were, additionally, 3D-Gaussian-filtered (Amira 5.2; FEI, Eindhoven, The Netherlands). Slices are shown in the corresponding voxel size thickness.

Live Cell Three-Dimensional Structured Illumination Microscopy - 3D-SIM

Our setup was equipped with a manufacturer-supplied apparatus and objective heater to allow medium-term imaging under standard live cell conditions (37 °C and 5 % CO_2). Previous live-samples assessed by 3D-SIM have concentrated on bacterial studies at 30 ºC (Strauss et al., 2012; Turnbull et al., 2014) or for HeLa cells at 24 ºC (Shao et al., 2011). The OMX V3 Blaze system parameters were adjusted for temperature-dependent changes in immersion oil refractive index with single layers of TetraSpeck fluorescent microspheres (Life Technologies), and optical transfer functions (OTFs) were generated from individual 110 nm diameter 505/515 fluorescent microspheres (Life Technologies). Phase parameters were adjusted for optimal imaging at 488 nm, and data was acquired using an appropriately matched immersion oil. 3D-SIM data were acquired with a Z-distance of 125 nm and with 15 images per plane (five phases, three angles). The raw data was computationally reconstructed using a Wiener (high-frequency) filter setting of 0.002 and channel- and temperature-specific OTFs employing the softWoRx 6.0 software package (GE Healthcare) to obtain a super-resolution 3D image stack of 32-bit depth, with a pixel size of 40x40 nm. Typically nuclei imaged were in between 6 µm and 12 µm in height. The resolution achieved was ~120 nm in the lateral direction and ~300 nm in the axial direction. The reconstructed data was thresholded for each channel to the stack modal grey value (representing the centre of the background intensity level) and converted to 16-bit tiff-stacks.

Correlative Fluorescence and Soft X-Ray Cryo-Microscopy/Tomography - CryoXM/T - at the HZB TXM at Beamline U41-FSGM of the BESSYII Electron Storage Ring in Berlin/Germany

Incubation of the BK cells on special grids for the Helmholtz-Zentrum Berlin full-field transmission soft X-ray microscope (HZB TXM), live-cell light microscopy, cryo-immobilization by plunge-freezing and correlated fluorescence/cryoXM/T are detailed in (Hagen et al., 2012) and (Hagen et al., 2014). For methodological reviews, see (Dent et al., 2014; Schneider et al., 2012).

Focused-Ion Beam Milling – CryoFIB - in a Dual Beam Scanning Electron FIB-SEM Cryo-Microscope

Owing to the lack of three-dimensional targeting methods (Fukuda et al., 2014), successful milling depends heavily on high-densities of the target structures. In nuclei of BK cells, required density of target structures had been confirmed by CEMOVIS sectioning. Vitreous samples on grids were mounted into modified Autogrids (FEI) to increase stability. These were transferred into a dual-beam FIB-SEM microscope (Quanta 3D FEG, FEI) by means of a cryo-transfer system (PP3000T, Quorum Technologies Ltd, Laughton, UK) and a custom-built transfer shuttle/sample holder (Rigort et al., 2010). During operation, samples were kept at a constant temperature below -180 °C using a homemade 360° rotatable cryo-stage (Rigort et al., 2012). To minimize curtaining artefacts during FIB milling, the sample was coated by organometallic platinum (GIS, FEI). Thin lamellae were prepared using Ga^{+} ions at 30 kV, under an effective beam to grid surface angle of 13°. Rough milling was performed with a rectangular pattern and 0.3 nA beam current, followed by sequentially lowered beam currents of 0.1 nA, 50 pA and 30 pA during the thinning and cleaning steps. Additionally, the specimen was observed by SEM at 5-10 kV and 5–50 pA.

Sub-Tomogram Averaging of CryoFIB/ET Data

The NEC units, forming a near continuous coat, did not provide clearly discernible features that might allow rational assignment of particle centres. Consequently, 31 vesicles from three tomograms were modelled as spheres to produce a series of 'particle' centres to be used for sub-tomogram averaging. The radius of each sphere was adjusted to the approximate mean radius of the NEC coat for each vesicle. Two out of three particle orientation parameters could be approximated using a 'particle axis' defined as normal to the sphere surface at each particle centre. Particle axes were then transformed to coincide with the Y-axis, and orientations around Y randomized to assist compensation of the 'missing wedge'. A spherically curved mask, 12 pixels (13.68 nm) thick, and 40 pixels wide was used to limit CCC to regions including protein signal, but not the vesicle membrane (VM). Tomograms were binned twice (to 11.4 Å/pixel), and initial averages calculated from a single vesicle of the highest quality and sphericalness over a cross-correlation box size of ~40 nm. This procedure involved a series of iterative CCC searches, in which translational search ranges were initially high, used a number of reference volumes selected from the vesicle itself as a reference template, and involved a full angular search around the particle axis (Y). For each starting reference, this approach resulted in averages showing pronounced hexagonal features with a centre-to-centre distance between unit cells (lattice spacing) of ~10 nm. Based on this observation, spherical sampling was optimized to oversample the NEC coat (nearest neighbour distance between points of ~3 nm). Sub-tomogram averaging was repeated for 9 vesicles after the initial result was centred on a 3-fold axis of symmetry and re-oriented to place the new particle axis on Y. To compensate for vesicle distortions that resulted in variable curvature of the NEC coat, a feature more pronounced for some vesicles than others, the CCC box was limited to a 26 pxl^3 box (~30 nm edge length), and an angular search range around Y of 120° was used. For each vesicle, cross-correlation searches were carried out over a number of iterations in which the angular range and step were made progressively finer, with final result being resubmitted for refinement using the same protocol until no improvements were observed. CCC was limited to a spatial frequency of 1/50 $Å^{-1}$ to ensure that alignment of noise did not significantly bias the alignment to the initial template. Duplicate particles (sharing particle centre coordinates) were removed after each iteration to produce an improved map for each vesicle from 200-350 particles. Final averages were calculated from 94 pxl^3 boxes and revealed that the NEC coat was in many cases ordered beyond the cross-correlated region. Additionally, the appearance of the VM, as well as other features in the map not involved in cross-correlation lent support to the validity of the alignment. Notably, while CCC searches were carried out about the 3-fold axes, the NEC averages exhibited 2-fold symmetry, and were consequently centred on the corresponding 2-fold axis, and the particle oriented around Y to place the *'6-2-6'* plane in coincidence with the X-axis. Owing to variability in curvature between vesicles – owing to size and shape - vesicle averages were not combined, nor was rotational symmetry applied to the vesicle maps at any stage. Resolution estimation was carried out for each vesicle

independently using the functions provided by PEET and did not follow the 'gold-standard' approach owing to the size of the vesicle particle datasets. Volumes were visualized in 3DMOD of IMOD (Kremer et al., 1996), or UCSF Chimera (Yang et al., 2012).

Construction of Soluble NEC Expression Vector

For expression of pU$_L$31 lacking the NLS (aa 26-271) and pU$_L$34 (aa 1-179) tagged with hexa-histidine (see Figure S3), the following primers were used to amplify the corresponding regions by PCR on genomic PrV strain Kaplan DNA as template:

U$_L$34-mpfc, CAC AGG ATC CGA CGA CCA TGA GCG GCA CC, BamHI, 31483-31502;
U$_L$34-mprc, CAC AAA GCT TGC GGG AGA CCG AGC GCT G, HindIII, 32027-32010;
U$_L$31-25F, CAC AGG TAC CGA TCG CTA CGC GCC CTA C, KpnI, 29507-29489 and
U$_L$31-mpr, CAC AGG TAC CCG GGC GAG GGG GGC GAA AG, KpnI, 28769-28787 with location numbers corresponding to Gene Bank Accession No. JQ809328.

Restriction enzyme sites were introduced for convenient cloning into corresponding restriction sites present in MCS1 (U$_L$34) and 2 (U$_L$31) of vector pETDuet-1 (Novagen). Correct amplification and insertion was verified by sequencing.

Small Angle X-Ray Scattering -SAXS - Data Collection, Processing, and Analysis

Data were collected from protein coming directly from a coupled size exclusion chromatography unit. The scattering patterns were measured in buffer B with 5 mM TCEP (tris(2-carboxyethyl)phosphine). The raw data were processed using PRIMUS (Konarev et al., 2003). The radii of gyration, R_g, of the particles were calculated from the Guinier approximation, as well as by use of the GNOM indirect transform package (Svergun, 1992), which calculated the distance distribution function *P*(r).

Ten independent *ab initio* models for the soluble NEC were computed with the simulated annealing *ab initio* bead modelling programs DAMMIF (Franke and Svergun, 2009). The ten models were aligned, averaged and filtered using the DAMAVER software suite (Volkov and Svergun, 2003). The model was further refined with DAMMIN (Svergun, 1999).

Architectural and SAXS-EM Integrative Model Building and Analysis

The sub-tomogram average is a 3.5-4-nm resolution representation of the NEC coat that does not unambiguously reveal the arrangement of heterodimers. The 'archway' and composing angular motifs are suggestive of a repeating feature that likely corresponded to this unit. Initially, we probed this assumption using a model of spheres corresponding to the approximate size of each component. Assuming a mass of ~30 kDa for both pU$_L$31 and pU$_L$34, each component of the NEC was initially modelled as a spherical volume ~3.5 nm in diameter. Guided by observation of lattice-type arrangements in the density, it was found that by placing these model spheres at the vertices of the lattices (Figure 4), a model could be devised which closely replicated the appearance of the experimental map for the NEC coat. The MD layer subunits were organized as clusters of 3 units (homotrimers). The placement of spheres reproduced and accounted for the 'angular' motif corresponding to a presumed heterodimer. The model subsequently served as the basis for the schematic shown in Figure 5. While this approach accounted for the 'pillars' of the 'archways', the nature of the 'arch' densities were initially difficult to reconcile, however the shared connection to the VM – also visible in raw tomograms - strongly suggests a homomeric interaction between pU$_L$34 in these regions with oligomerization taking place at sites distant (~3.2 nm) to the VM embedded C-terminal region (TM domain) – thereby forming the 'arch keystone'. Given the flexibility of the pU$_L$34 C-terminal part (Figure S3C), and that this is logically expected to be the closest part to the VM, the assertion was supported by our model. The quaternary model describes the hexagonal unit cell as consisting of a hexamer of pU$_L$31/34 heterodimers, and predicts at least three primary protein-protein interfaces contribute to assembly of the lattice in the first instance. These are the heterodimeric interface (pU$_L$31-pU$_L$34) which serves as the primary series of interfaces between the observed layers, as well as homomeric interactions of pU$_L$31 and pU$_L$34. Independently, pU$_L$31 was modelled initially

as a homotrimer, and these were placed on the vertices of the MD hexagonal lattice. It is equally possible that pU_L31 forms a homodimer in solution, with weaker interactions nucleating around the 3-fold axes resulting in coat formation. However, pU_L31 self-interaction in solution was not observed experimentally (Lorenz et al., 2015).

To investigate the orientation of soluble NEC within the cryoEM NEC coat unit cell – and move towards validation of the above model - we systematically cross-correlated $[pU_L31/34]_6$ SAXS models produced by varying the orientation of the heterodimer with respect to the cryoEM map. The SAXS PDB was converted to an EM density map (11.4 Å /pixel, 3 nm resolution) using the PDB2MRC program of the EMAN package (Tang et al., 2007). This map was centred on a 6-fold axis, oriented such that the NEC coat was perpendicular to Y-axis, and windowed to a 20 pxl^3 box. The SAXS map was thresholded tightly to produce an 'envelope' corresponding to a volume of ~87 nm^3. Thresholding served to approximately equalize internal densities such that these would not significantly influence the cross-correlation search against the cryoEM map (i.e. a fitting based on 'shape' alone was carried out). A series of hexameric models were created from $pU_L31/34$ envelopes using SPIDER by systematically varying six parameters (Shaikh et al., 2008). The first three parameters (phi, theta, psi) determined the orientation of the heterodimer within the unit cell, while translational parameters y, and x governed the displacement of the heterodimer from the NEC coat unit cell (particle average) centre along the Z- (unit cell radius) and Y- axis (vesicle radius), respectively. An angular step of 10° was used for the orientation search, while a step of 5.7 Å was used for the translation search. A hexameric model was created from the oriented and positioned envelope by populating heteordimers about the Y-axis according to 6-fold rotational symmetry. The final search parameter was a rotational search around the Y-axis, and was carried out over a 60° range (10° step) to identify each hexameric model best 'in-plane' orientation within the NEC coat unit cell. The SAXS-EM model supported interactions between components of the NEC coat proposed by the initial modelling (described above). Furthermore, the fit of the SAXS map allowed us to estimate the approximate number of amino acids contributing to each arch densities (C-terminal VM-connecting part of pU_L34 except the transmembrane domain) as: $pU_L34^{full\text{-}length}$-$pU_L34^{construct\text{-}length}$ (240-179) x 6 pU_L34 subunits/unit = 366 amino acids (~40.2 kDa). The sequence of pU_L34 is annotated in Figure S3C.

Modelling and Analysis of Curvature of the NEC Coat

Based on the observation of the two characteristic NEC layers (Figure 4), we hypothesized that the pattern of local interactions between components governs global properties of the vesicle by giving rise to 'well-defined' curvature when perpetuated over large surface areas; behaviour that should be amenable to modelling and analysis. The characteristic layers exhibited distinct hexagonal properties that could be represented by 'hypothetical lattices' for the MD and MP layers as shown in Figures 7A and S4. Serving as a convention for measurement, however, both layers correspond to a *p6* curved lattice. With respect to the integrative SAXS model, the MP and MD layers are positioned at either radial side of the heterodimer core ('pillar'); i.e. these coincide with locations where homomeric interactions are established and act to spatially constrain the placement of each subunit. Attempts to overlay the hypothetical lattices, i.e. as a geometric model of the NEC coat, showed these do not overlap precisely over distances larger than that of the unit of the MD lattice (Figure 7A). However, if we constrained the hexagonal unit centres of the shared unit cell to overlap (as would be carried out by multiple instances of macromolecular association), we find that precise curvature allows for radial alignment of the units (as shown in Figure 7B). The model suggested further that heteromeric association is required for curvature, and that these protein-protein interfaces are organized about the shared 2-fold axes of each layer (this was supported by SAXS-EM modelling, Figure 6). Notably, the geometric model predicts that it is this location of the heteromeric interactions that establishes the *'6-2-6'* directionality of curvature as observed in the experimental map (Figure 5 shows that the MD layer is – unexpectedly - planar between *'3-2-3'* axes), and consequently the modelling described below was carried out for the *'6-2-6'* cross-sectional plane.

To model curvature of the NEC coat, we derived an equation relating structural properties of the NEC to the radius to, or diameter between, the membrane distal layer(s) (MD) of the vesicle NEC coat (r_{MD} and d_{MD}, respectively). Equation 1 describes these in relation to the quotient ('*a*') of two arc lengths (V_{MP} and V_{MD}) between two adjacent 2-fold axes that are separated radially by a distance '*h*' (Figure 7).

$r_{MD}=h/(a\text{-}1)$, where $a=V_{MP}/V_{MD}$ (Equation 1)

The model assumes that the NEC coat achieves near complete coverage of the vesicles inner surface; an assumption supported by inspection of the tomograms. Conversely, use of the formula to successfully predict vesicle size based on local measurements from the NEC sub-tomogram average would serve to support that near complete coverage of the vesicle inner surface by the NEC coat does take place, and that the NEC coat architecture is perpetuated over most of this surface area. Sufficiently accurate measurement of these parameters should allow the appearance of the NEC coat to be modelled, as illustrated in Figure 7. *'h'* (~5.13 nm) is defined as the distance between distinct hexagonal arrangements of the MD and MP layers – coinciding with the 'pillars' of the archway, and '*a*' calculated as the quotient between arch lengths (as approximated from lattice spacing) between layers. The overall scheme for these measurements and respective calculations are described in Figure S4. To prepare for these measurements the NEC map was interpolated by a factor of two (5.7 Å/pixel), and tangential slices masked to include only well-defined unit cells, and subsequently padded by a factor of six to oversample the Fourier transform. r_{MD} (e.g. ~58.6 nm for the vesicle featured) can be measured from the experimental average in relation to the spherical model used for averaging, or as illustrated in Figures 7 and S4, calculated (predicted) from measurements taken locally from the experimental NEC coat average map. To scale arc lengths so as to construct the model overlay shown in Figure 7B, a radial projection of the NEC average was calculated using the BRADIAL program of BSOFT (Heymann et al., 2008), and this allowed the true magnitude of unit cell spacing (V_{MD}) to be measured. To produce the 2D *'6-2-6'* model overlay a hexagonal unit was created by modelling a heterodimer (the 'angular motif'), this was then displaced by half the measured V_{MD} with a reflection about the vertical axis to produce a unit cell cross-section. This unit model was then used to produce entire vesicle extrapolations by applying the angle subtended by the arc lengths measured from the sub-tomogram averages. This approach simulated the influence of the pU_L34 'arch', in spatially constraining the position of the heterodimer 'pillars'. According to this model the MD layer is thus formed by 2-fold and 3-fold interactions of pU_L31, while the MP layer varies with curvature, but is formed primarily by the 6-fold 'arch interactions', but also by interactions occurring on the 2-fold axes. The model predicts that complete coverage of the vesicle inner surface is geometrically allowed, as shown in Figure 7D, which is an extrapolation of that shown in Figure 7B.

SUPPLEMENTAL REFERENCES

Al-Amoudi, A., Studer, D., and Dubochet, J. (2005). Cutting artefacts and cutting process in vitreous sections for cryo-electron microscopy. J. Struct. Biol. *150*, 109-121.

Bleck, C.K.E., Merz, A., Gutierrez, M.G., Walther, P., Dubochet, J., Zuber, B., and Griffiths, G. (2010). Comparison of different methods for thin section EM analysis of *Mycobacterium smegmatis*. J. Microsc. *237*, 23-38.

Bouchet-Marquis, C., and Hoenger, A. (2011). Cryo-electron tomography on vitrified sections: a critical analysis of benefits and limitations for structural cell biology. Micron *42*, 152-162.

Dent, K.C., Hagen, C., and Grünewald, K. (2014). Critical step-by-step approaches toward correlative fluorescence/soft X-ray cryo-microscopy of adherent mammalian cells. In Correlative Light and Electron Microscopy II, T. Müller-Reichert, and P. Verkade, eds. (Burlington, MA: Academic Press), pp. 179-216.

Fukuda, Y., Schrod, N., Schaffer, M., Feng, L.R., Baumeister, W., and Lučić, V. (2014). Coordinate transformation based cryo-correlative methods for electron tomography and focused ion beam milling. Ultramicroscopy *143*, 15-23.

Gunkel, M., Schöneberg, J., Alkhaldi, W., Irsen, S., Noe, F., Kaupp, U.B., and Al-Amoudi, A. (2015). Higher-order architecture of rhodopsin in intact photoreceptors and its implication for phototransduction kinetics. Structure *23*, 628-638.

Hagen, C., Werner, S., Carregal-Romero, S., Malhas, A.N., Klupp, B.G., Guttmann, P., Rehbein, S., Henzler, K., Mettenleiter, T.C., Vaux, D.J., *et al.* (2014). Multimodal nanoparticles as alignment and correlation markers in fluorescence/soft X-ray cryo-microscopy/tomography of nucleoplasmic reticulum and apoptosis in mammalian cells. Ultramicroscopy *146*, 46-54.

Han, H.M., Zuber, B., and Dubochet, J. (2008). Compression and crevasses in vitreous sections under different cutting conditions. J. Microsc. *230*, 167-171.

Heymann, J.B., Cardone, G., Winkler, D.C., and Steven, A.C. (2008). Computational resources for cryo-electron tomography in Bsoft. J. Struct. Biol. *161*, 232-242.

Hsieh, C.E., Leith, A., Mannella, C.A., Frank, J., and Marko, M. (2006). Towards high-resolution three-dimensional imaging of native mammalian tissue: electron tomography of frozen-hydrated rat liver sections. J. Struct. Biol. *153*, 1-13.

Konarev, P.V., Volkov, V.V., Sokolova, A.V., Koch, M.H.J., and Svergun, D.I. (2003). PRIMUS: a Windows PC-based system for small-angle scattering data analysis. J. Appl. Crystallogr. *36*, 1277-1282.

Kremer, J.R., Mastronarde, D.N., and McIntosh, J.R. (1996). Computer visualization of three-dimensional image data using IMOD. J. Struct. Biol. *116*, 71-76.

Malhas, A., Goulbourne, C., and Vaux, D.J. (2011). The nucleoplasmic reticulum: form and function. Trends Cell Biol. *21*, 362-373.

Mastronarde, D.N. (2008). Correction for non-perpendicularity of beam and tilt axis in tomographic reconstructions with the IMOD package. J. Microsc. *230*, 212-217.

Mielanczyk, L., Matysiak, N., Michalski, M., Buldak, R., and Wojnicz, R. (2014). Closer to the native state. Critical evaluation of cryo-techniques for Transmission Electron Microscopy: preparation of biological samples. Folia Histochem. Cytobiol. *52*, 1-17.

Pierson, J., Ziese, U., Sani, M., and Peters, P.J. (2011). Exploring vitreous cryo-section-induced compression at the macromolecular level using electron cryo-tomography; 80S yeast ribosomes appear unaffected. J. Struct. Biol. *173*, 345-349.

Rigort, A., Bäuerlein, F.J.B., Leis, A., Gruska, M., Hoffmann, C., Laugks, T., Böhm, U., Eibauer, M., Gnaegi, H., Baumeister, W., *et al.* (2010). Micromachining tools and correlative approaches for cellular cryo-electron tomography. J. Struct. Biol. *172*, 169-179.

Schneider, G., Guttmann, P., Rehbein, S., Werner, S., and Follath, R. (2012). Cryo X-ray microscope with flat sample geometry for correlative fluorescence and nanoscale tomographic imaging. J. Struct. Biol. *177*, 212-223.

Shaikh, T.R., Gao, H.X., Baxter, W.T., Asturias, F.J., Boisset, N., Leith, A., and Frank, J. (2008). SPIDER image processing for single-particle reconstruction of biological macromolecules from electron micrographs. Nat. Protoc. *3*, 1941-1974.

Shao, L., Kner, P., Rego, E.H., and Gustafsson, M.G.L. (2011). Super-resolution 3D microscopy of live whole cells using structured illumination. Nat. Methods *8*, 1044-1046.

Sorzano, C.O.S., Messaoudi, C., Eibauer, M., Bilbao-Castro, J.R., Hegerl, R., Nickell, S., Marco, S., and Carazo, J.M. (2009). Marker-free image registration of electron tomography tilt-series. BMC Bioinformatics *10*, 124.

Svergun, D.I. (1992). Determination of the regularization parameter in indirect-transform methods using perceptual criteria. J. Appl. Crystallogr. *25*, 495-503.

Svergun, D.I. (1999). Restoring low resolution structure of biological macromolecules from solution scattering using simulated annealing. Biophys. J. *76*, 2879-2886.

Tang, G., Peng, L., Baldwin, P.R., Mann, D.S., Jiang, W., Rees, I., and Ludtke, S.J. (2007). EMAN2: An extensible image processing suite for electron microscopy. J. Struct. Biol. *157*, 38-46.

Turnbull, L., Strauss, M.P., Liew, A.T.F., Monahan, L.G., Whitchurch, C.B., and Harry, E.J. (2014). Super-resolution imaging of the cytokinetic Z ring in live bacteria using fast 3D-structured illumination microscopy (f3D-SIM). J. Vis. Exp. *91*, e51469.

Volkov, V.V., and Svergun, D.I. (2003). Uniqueness of *ab initio* shape determination in small-angle scattering. J. Appl. Crystallogr. *36*, 860-864.

Yang, Z., Lasker, K., Schneidman-Duhovny, D., Webb, B., Huang, C.C., Pettersen, E.F., Goddard, T.D., Meng, E.C., Sali, A., and Ferrin, T.E. (2012). UCSF Chimera, MODELLER, and IMP: an integrated modeling system. J. Struct. Biol. *179*, 269-278.
